# Rab4b controls hepatic glucose production during fasting through glycophagy

**DOI:** 10.1038/s41467-026-76218-8

**Published:** 2026-08-10

**Authors:** Marion Dussot, Lucie Le Parc, Sihame Sabbane, Alycia Zedda, Abderrahman Chafik, Alexandre Gallerand, Karine Dumas, Stéphanie Bonnafous, Yun Kwon, Sandra Lacas-Gervais, Charlotte Hinault Boyer, Giulia Chinetti, Sophie Giorgetti-Peraldi, Philippe Gual, Stoyan Ivanov, Anja Zeigerer, Jean-François Tanti, Mireille Cormont, Jerome Gilleron

**Affiliations:** 1 Université Côte d’Azur, INSERM U1065, Adipo-Cible Research Study Group, Mediterranean Center of Molecular Medicine (C3M), Team “Insulin Resistance in Obesity and Type 2 Diabetes”, Nice, France; 2 Université Côte d’Azur, CNRS, Adipo-Cible Research Study Group, LP2M, Team “Cellules myéloïdes et métabolisme”, Nice, France; 3 Université Côte d’Azur, CHU, INSERM U1065, Adipo-Cible Research Study Group, Mediterranean Center of Molecular Medicine (C3M), Team “Chronic liver diseases associated with obesity and alcohol “, Nice, France; 4 European Center for Angioscience (ECAS), Medical Faculty Mannheim, Heidelberg University, Mannheim, Germany; 5 Institute for Diabetes and Cancer, Helmholtz Center Munich, 85764 Neuherberg, Germany, Joint Heidelberg-IDC Translational Diabetes Program, Inner Medicine 1, Heidelberg University Hospital, Heidelberg, Germany; 6 German Center for Diabetes Research (DZD), Neuherberg, Germany; 7 Université Côte d’Azur, CCMA, Centre Commun de Microscopie Appliquée (CCMA), Nice, France; 8 Université Côte d’Azur, Centre Hospitalier Universitaire de Nice, Laboratoire de Biochimie-Hormonologie, Hôpital Pasteur, Nice, France; 9 Université Côte d’Azur, INSERM U1065, Mediterranean Center of Molecular Medicine (C3M), Team “Cancer, Métabolisme et Environnement”, Nice, France

**Keywords:** Homeostasis, Macroautophagy, Small GTPases

## Abstract

Hepatic glucose production, essential for maintaining glycemia, is often altered in metabolic-associated fatty liver and glycogen storage diseases. Therefore, understanding the mechanisms that regulate the glucose production by hepatocyte is pivotal. Recent studies have identified endocytic trafficking as a key modulator of liver glucose production. Particularly, its role in directing protein trafficking toward lysosomal degradation influences gluconeogenesis. However, the contribution of endocytic recycling to liver glucose production remains yet poorly understood. Here, we demonstrate that hepatocyte-specific disruption of endocytic recycling, achieved through Rab4b knockout in male, leads to fasting hyperglycemia. Mechanistically, hepatocytes lacking Rab4b exhibit an increased capacity to produce glucose independently of gluconeogenesis by depleting their glycogen stores. This is driven by enhanced glycophagy, leading to excessive glucose release and fasting hyperglycemia. Notably, liver Rab4b expression also correlates with glycemia in both mice and human, highlighting a critical role of Rab4b-dependent endocytic recycling in regulating blood glucose homeostasis during fasting.

## Abstract (web summary)

This study reveals an unexpected role for Rab4b-dependent endocytic recycling in orchestrating the liver’s response to fasting, linking autophagy-driven glycogen breakdown to glucose production.

## Introduction

The liver is a pivotal organ for glucose homeostasis accounting for ~90% of endogenous glucose production^1^. This function is fulfilled by the capacity of hepatocytes to store postprandially the excess of blood glucose and to produce glucose during fasting^2,3^. Indeed, in the fasting state, glucagon signaling within hepatocytes induces i) the expression of rate-limiting enzymes of gluconeogenesis to produce glucose from substrates such as lactate, pyruvate and glycerol^2,4^; ii) glycogenolysis to break-down glycogen by activating glycogen phosphorylase^2^; and iii) an autophagic process of glycogen degradation called glycophagy^5^. Glycophagy is initiated by the recruitment of glycogen to the phagophore by the glycogen receptor Stbd1. Glycogen granules that reach the autolysosomes are then degraded as glucose by the lysosomal enzyme acid α-glucosidase (Gaa)^6^. The contribution of glycophagy to liver glucose production is still debated. Indeed, although glycophagy in newborns is crucial to fight neonatal hypoglycemia by producing glucose from glycogen in response to glucagon stimulation^7^, its implication in adults remains unclear.

Endocytic trafficking is a cellular process by which receptors and nutrients internalized by endocytosis are sorted from early endosomes toward either the degradative routes through late endosomes and lysosomes, or recycled back to the plasma membrane through recycling endosomes^8^. There is now in vivo evidence that endocytic-dependent degradation is an important regulator of hepatic glucose metabolism^9,10^. Indeed, restraining all receptors signaling at the plasma membrane by depleting the entire endolysosomal system in mouse liver by silencing Rab5, reduces liver glucose production by suppressing gluconeogenesis^11,12^. Moreover, blocking endocytic degradation leads to glucagon receptor accumulation in early endosomes, increasing gluconeogenesis^13^. Taken together, these studies have highlighted the role of endocytic degradative pathway in liver glucose production. However, to our knowledge, the role of endocytic recycling pathways in vivo on liver glucose production is ill-defined.

Endocytic recycling is governed by members of the Rab GTPase family^14^. Among them, the Rab4 sub-family is critical for the early stages of endocytic recycling pathways^15,16^. We have previously shown that within this subfamily, the small GTPase Rab4b controls several endocytic recycling pathways^17,18^. Moreover, we evidenced that Rab4b plays a key role in metabolism. Indeed, Rab4b silencing in vitro increases glucose uptake by favoring the plasma membrane translocation of glucose transporter Glut4 in adipocytes^19^. In addition, we found that Rab4b is downregulated in white adipose tissue T cells from patients with obesity and insulin resistance, and that its depletion in T cells in vivo leads to adipose tissue dysfunction and insulin resistance on chow diet^20^. However, whether Rab4b-dependent endocytic recycling pathways regulates liver metabolism is currently unknown.

Here, we show that specific depletion of Rab4b in hepatocytes in vivo alters endocytic recycling pathways. We therefore used this genetic model as a tool to address the important question of the role of endocytic recycling in liver metabolism. Our study shows that Rab4b depletion reprograms the metabolic transcriptome during fasting. Mechanistically, we found that hepatocytes lacking Rab4b produce more glucose through glycophagy, resulting in transient fasting hyperglycemia. These findings reveal a unique role of Rab4b in glycophagy regulation, highlighting a previously unrecognized role of Rab4b-dependent endocytic recycling in glucose homeostasis.

## Results

### **Hepatic Rab4b expression is regulated by nutritional status and correlates with blood glucose levels**

**Fig. 1:**
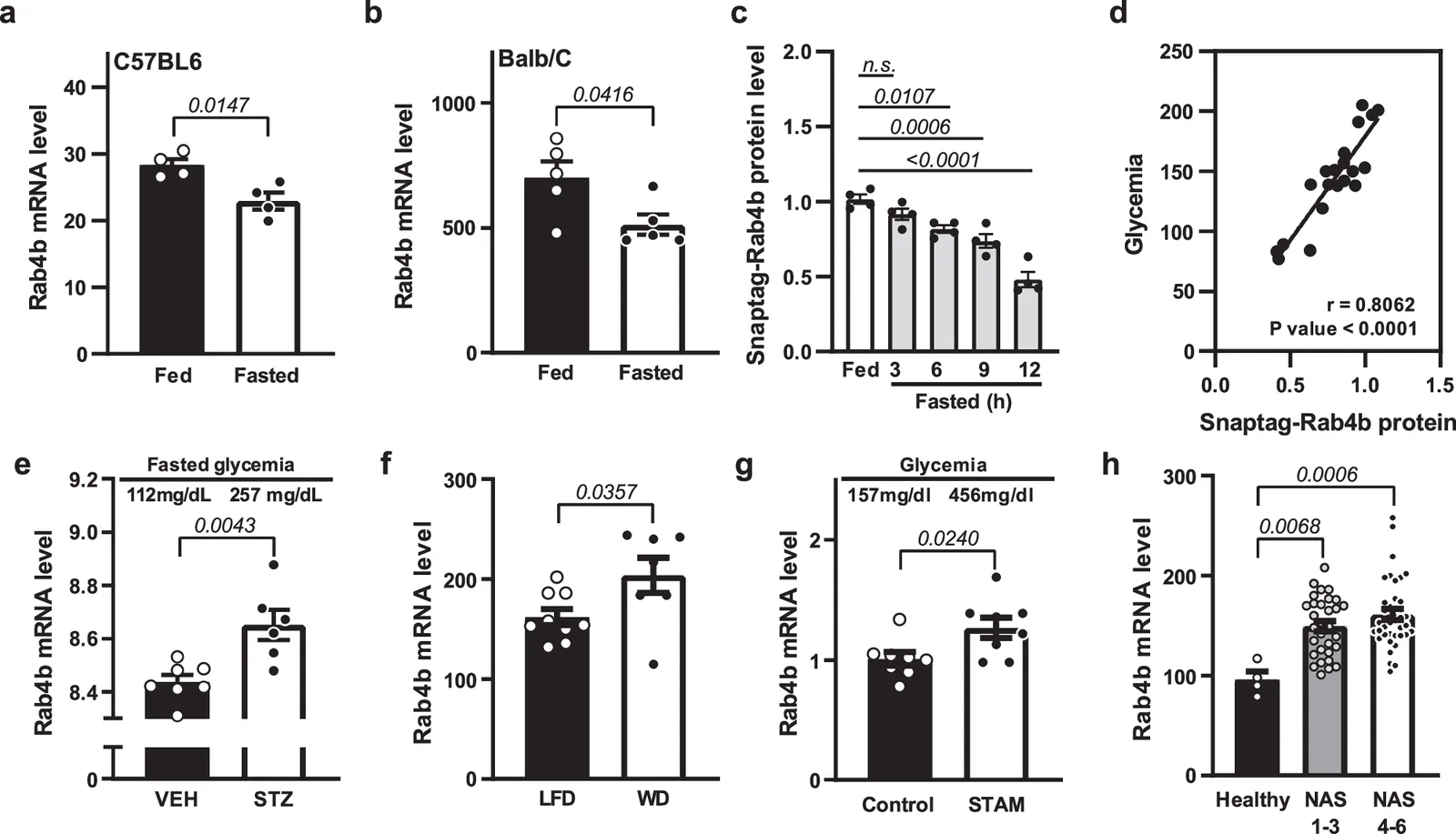
Rab4b expression correlates with glycemia. **a** *Rab4b* mRNA expression (Fragments Per Kilobase Million) in the liver of C57/Bl6 Rab4b^fl/fl^ mice either random-fed or fasted for 6 h (*n* = 4/genotype). **b** *Rab4b* mRNA expression in liver of Balb/C mice either fed or fasted, extracted from a published dataset GSE137385^68^ (*n* = 5/genotype). **c** Rab4b protein level in liver of C57/Bl6 Snaptag-Rab4b mice random-fed or during fasting (*n *= 4/conditions). **d** Correlation between Rab4b protein level and glycemia in fasted and fed C57/Bl6 Snaptag-Rab4b mice (*n* = 20). **e** *Rab4b* mRNA expression in liver from control (VEH – vehicle – *n* = 7) or streptozotocin (STZ – *n* = 6) treated mice, extracted from a published dataset GSE39752^21^. **f** *Rab4b* mRNA expression in liver from mice fed with a low-fat diet (LFD – *n* = 9) or a western diet (WD – *n* = 7), extracted from a published dataset GSE110404^23^. **g** *Rab4b* mRNA expression in liver from control or STAM mice (streptozotocin + high fat diet) (*n* = 8/genotype). **h** *Rab4b* mRNA expression in liver from healthy patients (*n* = 4), patients with a NAFLD Activity Score (NAS score) between 1 and 3 (*n* = 31 – NAFLD), or patients with a NAS score between 4 and 6 (*n* = 38 – NASH), extracted from a published dataset GSE130970^69^. Unpaired two-sided t-test was used for panels (**a**, **b**, **e**–**g**), one-way ANOVA with Sidak multiple-comparison correction for panels (**c**, **h**), and Pearson’s correlation with two-tailed *p*-values for panel (**d**). Error bars indicate means ± s.e.m. Source data are provided as a Source Data file.

Liver metabolism undergoes profound changes during the transition from fasting to feeding. To determine whether hepatic Rab4b expression is regulated during this transition, we compared *Rab4b* mRNA levels in the liver of mice fasted for 6 h and of randomly fed mice. As expected, 6 h of fasting lowered blood glucose levels and triggered a liver Pparα-dependent transcriptional response (Supplementary Fig. 1a–d). In addition, expression of the key glycolytic enzyme, glucokinase, was reduced in the liver of fasted compared to fed mice (Supplementary Fig. 1e). Notably, *Rab4b* mRNA levels were significantly lower in the liver of both C57Bl6 and Balb/C mice after 6 h of fasting compared to their randomly fed counterparts (Fig. 1a, b). Furthermore, hepatic *Rab4b* mRNA expression positively correlated with blood glucose levels but showed no association with body weight or fat mass (Supplementary Fig. 2a–c). To accurately assess endogenous Rab4b protein levels, we generated a SnapTag-Rab4b knock-in mouse model, as no selective anti-Rab4 antibodies were able to reliably detect endogenous Rab4b levels in the liver. Using this mouse model, we observed that the protein level of Rab4b began to significantly decrease after 6 h of fasting, reaching approximately a 50% reduction after 12 h (Fig. 1c and Supplementary Fig. 2d). As for the mRNA, the protein level of Rab4b is positively correlated with blood glucose levels (Fig. 1d). Strikingly, *Rab4b* mRNA expression was also significantly higher in the liver of several mouse models with hyperglycemia, including the streptozotocin-induced type-1 diabetes, western diet-induced obesity, and streptozotocin plus high-fat diet-induced-metabolic-associated steatohepatitis (MASH)^21–23^ (Fig. 1e–g). Similarly, in human liver, *RAB4B* mRNA expression was significantly higher in individuals with elevated NAFLD Activity Scores (NAS), which are known to correlate positively with blood glucose levels^24^ (Fig. 1h and Supplementary Fig. 2e).

Collectively, these results highlight a regulation of Rab4b expression in the liver during the fasting-to-feeding transition and suggest that a variation in glycemia may be a key driver of this regulation.

### Rab4b depletion in hepatocytes alters endocytic recycling

**Fig. 2:**
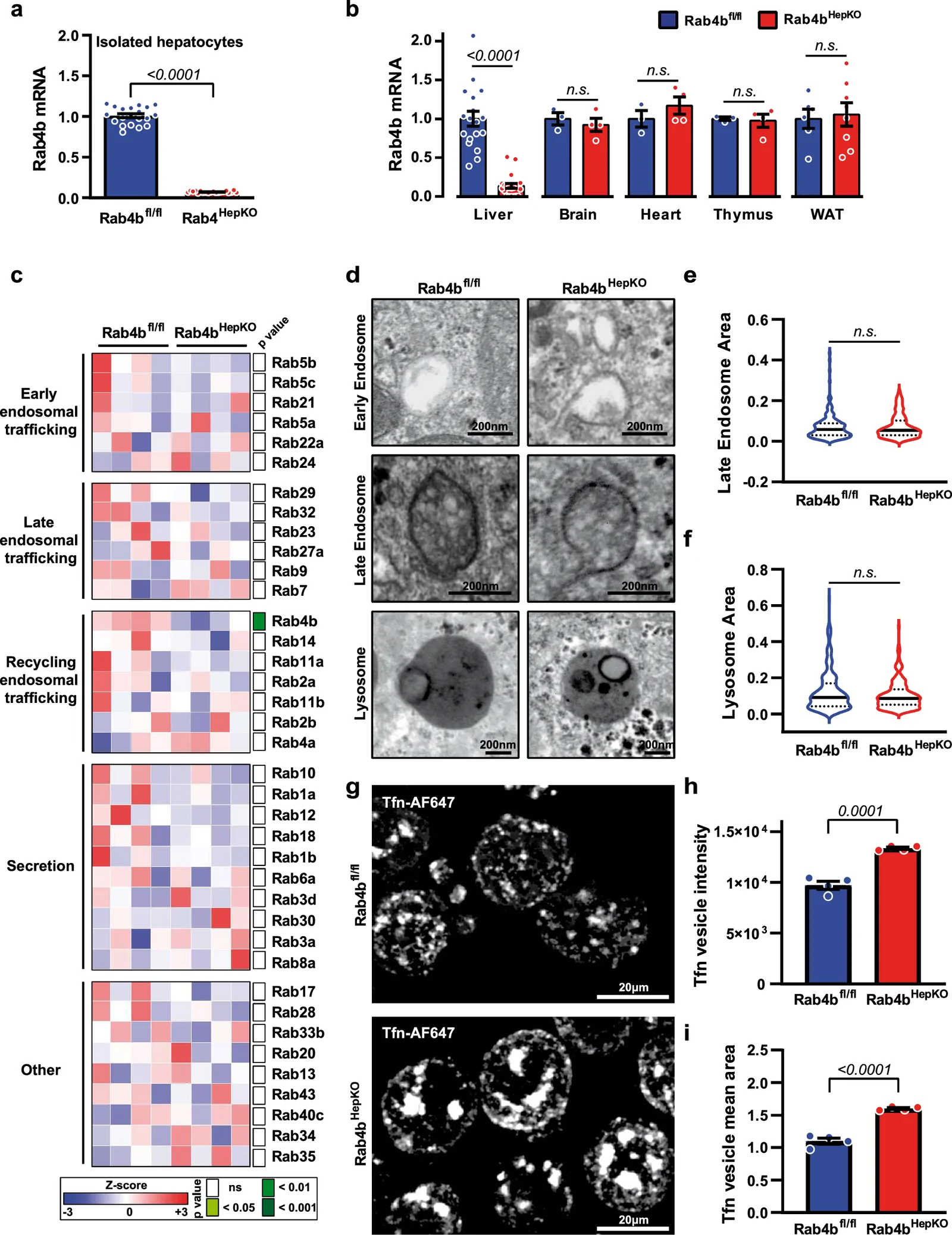
Rab4b depletion in mice hepatocyte in vivo impairs endocytic recycling function. **a** *Rab4b* mRNA expression in primary mouse hepatocytes isolated from Rab4b^fl/fl^ (control) and Rab4b^HepKO^ mice. mRNA levels are relative to mouse Rplp0. (*n* = 20/genotype). **b** *Rab4b* mRNA expression in Brain, Heart, Thymus, White Adipose Tissue (WAT), and Liver from Rab4b^fl/fl^ mice and Rab4b^HepKO^ mice. mRNA levels are relative to mouse Rplp0. (*n* = 3 Rab4b^fl/fl^ and 4 Rab4b^HepKO^ mice for Brain, Heart and Thymus; *n* = 6 Rab4b^fl/fl^ and 8 Rab4b^HepKO^ mice for WAT; *n* = 18 Rab4b^fl/fl^ and 21 Rab4b^HepKO^ mice for Liver). **c** mRNA levels of endocytic Rab proteins in liver from random-fed Rab4b^fl/fl^ mice and Rab4b^HepKO^ mice (*n* = 4/genotype). **d** Representative electron microscopy images of early endosomes, late endosomes and lysosomes in liver section from random-fed Rab4b^fl/fl^ mice and Rab4b^HepKO^ mice (*n* = 4/conditions). All scale bars represent 200 nm. **e** Quantification of late endosome area (µm^2^) obtained by manual segmentation (*n* = 165 for Rab4b^fl/fl^ and 138 for Rab4b^HepKO^ mice). **f** Quantification of lysosome area (µm^2^) obtained by manual segmentation (*n* = 210 for Rab4b^fl/fl^ and 170 for Rab4b^HepKO^ mice). **g** Representative light microscopy images of primary mouse hepatocytes freshly isolated from random-fed Rab4b^fl/fl^ and Rab4b^HepKO^ liver and incubated for 15 min with fluorescently labeled transferrin. (*n* = 4/genotype). **h** Quantification of transferrin-positive vesicle intensity (*n* = 4/genotype). **i** Quantification of transferrin-positive vesicle mean area (*n* = 4/genotype). Unpaired two-sided t-test with Welch’s correction was used for panel (**a**), one-way ANOVA with Kruskal-Wallis multiple-comparisons for panel (**b**), two-sided moderated t-tests using limma for panel (**c**), unpaired two-sided Mann-Whitney test for panels (**e**, **f**), and unpaired two-sided t-test for panels (**h**, **i**). Error bars indicate means ± s.e.m. Source data are provided as a Source Data file.

Given that Rab4b expression is modulated during the fasting-to-feeding transition, we hypothesized that endocytic recycling, a process in which Rab4b plays a key regulatory role^17,18^, may be involved in the metabolic adaptation of the liver to nutrient availability. To explore this, we generated hepatocyte-specific *Rab4b* knockout mice (Rab4b^HepKO^) by crossing Rab4b-floxed mice (Rab4b^fl/fl^ mice)^20^ with Alb-Cre mice, which express Cre recombinase under the control of the liver-specific albumin promoter (Supplementary Fig. 3a, b). *Rab4b* mRNA levels were reduced by 90% in liver and primary hepatocytes of Rab4b^HepKO^ mice compared to Rab4b^fl/fl^ mice, while expression in other tissues, including brain, heart, thymus, and white adipose tissue, remained unchanged (Fig. 2a, b). Rab4b^HepKO^ mice were healthy with no difference in body weight and food intake compared to Rab4b^fl/fl^ littermates (Supplementary Fig. 4a, b). Liver histology revealed no signs of tissue damage (Supplementary Fig. 4c), and the abundance of the different non-parenchymal cell populations was comparable between Rab4b^HepKO^ and Rab4b^fl/fl^ mice (Supplementary Fig. 5). Moreover, RNA-seq analysis of liver revealed that Rab4b depletion did not alter the expression of other Rab family members (Fig. 2c), ruling out compensatory transcriptional responses from other closely related Rab proteins.

We next examined the impact of hepatic Rab4b depletion on endocytic compartments and endocytic recycling. Transmission electron microscopy analyses of liver sections showed no alterations in the density or morphology of early endosomes, late endosomes, or lysosomes in Rab4b^HepKO^ mice compared to Rab4b^fl/fl^ mice (Fig. 2d–f and Supplementary Fig. 6a–g). To assess endocytic recycling function, we measured steady-state uptake of fluorescently labeled transferrin (Tfn), a canonical cargo of the endocytic recycling pathway^25^, in primary hepatocytes isolated from Rab4b^fl/fl^ and Rab4b^HepKO^ mice (Fig. 2g). Rab4b-deficient hepatocytes exhibited significantly increased uptake of fluorescent Tfn (Fig. 2g, h), consistent with our previous findings in HeLa cells^17^. This was associated with an increase in the number, the area, and the clustering of transferrin-positive structures (Fig. 2i and Supplementary Fig. 6h, i).

Collectively, these results demonstrate that depletion of Rab4b in hepatocytes modifies endocytic recycling function without affecting the endocytic compartment morphology.

### Rab4b depletion in hepatocytes reprograms their metabolic transcriptome

**Fig. 3:**
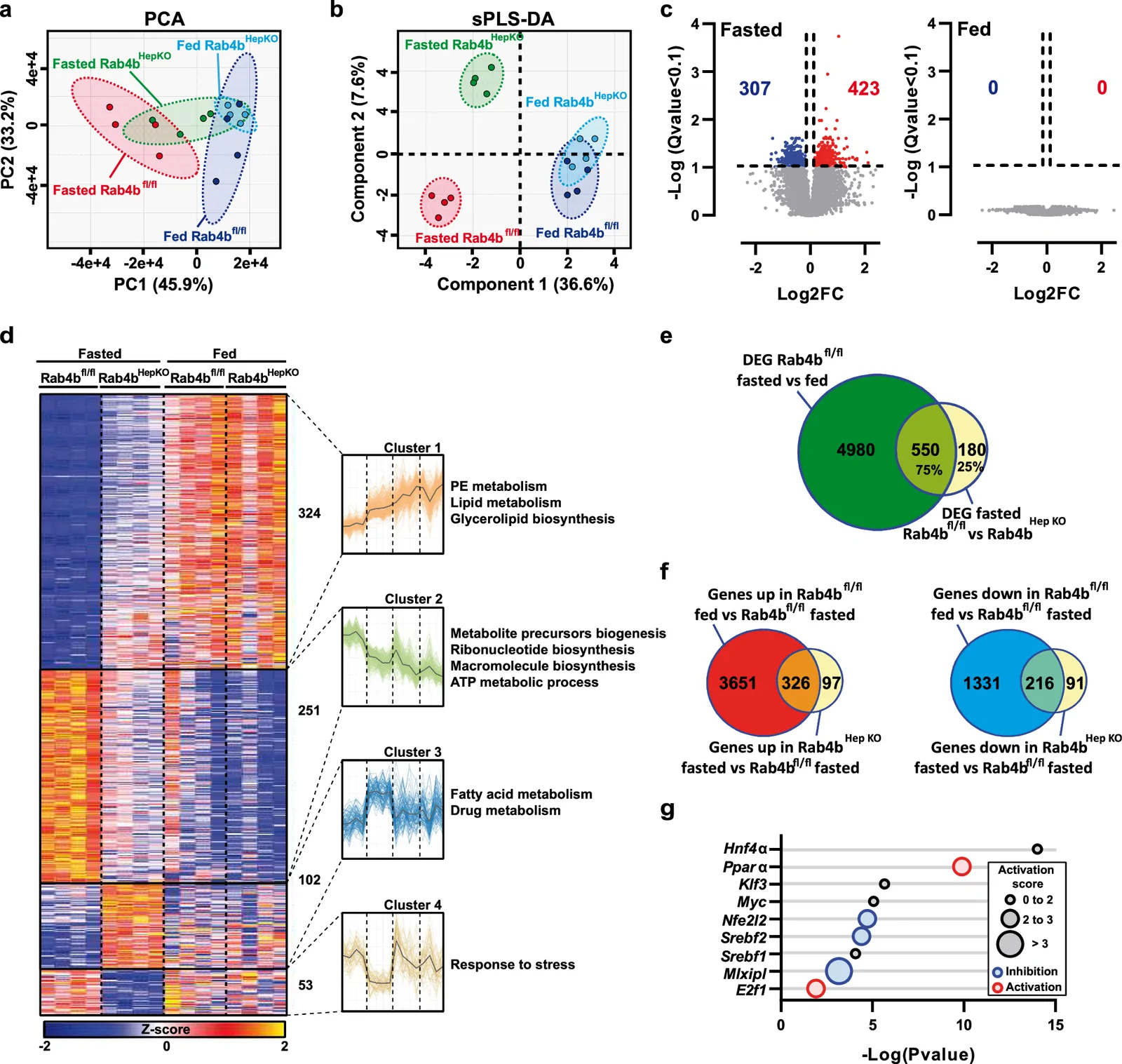
Rab4b depletion in mice hepatocyte in vivo strongly reprogram liver transcriptome upon fasting. **a**, **b** Principal Component Analysis (**a**) and sparse Partial Least Squares regression Discrimination Analysis (**b**) of liver transcriptome from control Rab4b^fl/fl^ (red & blue) and Rab4b^HepKO^ (green & cyan) mice upon fasting (red & green) or feeding (blue & cyan). Statistical confidence ellipses are represented in corresponding colors. **c** Volcano plot representing the genes significantly (Adjust *P* < 0.1) down-regulated (blue) and up-regulated (red) at the mRNA level in livers from Rab4b^HepKO^ mice compared to Rab4b^fl/fl^ mice upon fasting (left panel) and feeding (right panel). **d** Clustering of the differentially expressed genes considering genotypes and nutritional conditions. Inserts show single gene expression (colored lines) and mean expression (black line) for each cluster. GOrilla functional enrichment is written on the right of each insert. Data are presented as mean values +/- SD. **e** Venn Diagram combining genes differentially expressed between fasting and feeding conditions in control mice (green), with genes differentially expressed between Rab4b^fl/fl^ and Rab4b^HepKO^ mice upon fasting (yellow). **f** Left panel: Venn Diagram combining genes upregulated in fed Rab4b^fl/fl^ mice when compared to fasted Rab4b^fl/fl^ mice (red), with genes upregulated in fasted Rab4b^HepKO^ mice when compared to fasted Rab4b^fl/fl^ mice (yellow). Right panel: Venn Diagram combining genes downregulated in fed Rab4b^fl/fl^ mice when compared to fasted Rab4b^fl/fl^ mice (blue), with genes downregulated in fasted Rab4b^HepKO^ mice when compared to fasted Rab4b^fl/fl^ mice (yellow). **g** Transcription factors involved in fasting or feeding metabolic regulation identified by Ingenuity Pathway Analysis upstream regulators analysis. Transcription factors predicted to be activated are in red, and predicted to be repressed are in blue. Activation/inactivation scores are represented by the size of circles. Two-sided moderated t-tests using limma was used for panel (**c**). Source data are provided as a Source Data file.

To investigate the global impact of defective endocytic recycling in hepatocyte, we conducted a RNA-seq analysis on liver samples from both genotypes under fasted and fed conditions. Principal component analysis (PCA) revealed distinct transcriptomic profiles between fasted and fed mice in Rab4b^fl/fl^ mice (Fig. 3a). When comparing the two genotypes, their transcriptomes overlapped under fed conditions (*P* = 0.28). However, during fasting, the transcriptome of Rab4b^HepKO^ mice was significantly different from that of Rab4b^fl/fl^ mice and instead partially resembled the transcriptomic profile of fed mice. This difference was further confirmed by sparse partial least squares discriminant analysis (sPLS-DA), which showed a clear separation between the genotypes under fasting, while no such difference was observed under fed conditions (Fig. 3b). Consistent with these observations, 730 genes were differentially expressed (adjusted *P* < 0.1; 423 upregulated and 307 downregulated) in the liver of fasted Rab4b^HepKO^ mice compared to Rab4b^fl/fl^ mice (Fig. 3c). In contrast, no significant gene expression changes were observed under fed conditions (Fig. 3c), indicating that Rab4b depletion in hepatocytes specifically alters the liver transcriptome during fasting.

K-means clustering of these 730 differentially expressed genes identified four clusters (Fig. 3d). Intriguingly, these clusters were enriched for genes involved in metabolic processes. Cluster 1 (C1) included 324 genes associated with the metabolism of lipids, glycerolipid, and phosphatidylethanolamine. Cluster 2 (C2) comprised 251 genes related to metabolite and energy precursor biosynthesis, as well as ribonucleotide and macromolecule biosynthesis. Cluster 3 (C3) contained 102 genes related to fatty acid and drug metabolism, while Cluster 4 (C4) consisted of 53 genes linked to stress responses (Fig. 3d). There was no difference in gene expression in each cluster in the fed state between genotypes. However, under fasting, C1 and C3 were enriched in genes with an increased expression in the liver of Rab4b^HepKO^ mice compared to Rab4b^fl/fl^ mice, while C2 and C4 were enriched in downregulated genes. C1 and C2 included genes that responded to the fasting-to-feeding transition in the liver of Rab4b^fl/fl^ mice. Notably, of the 730 genes altered by the depletion of Rab4b in the fasting state, 75 % (550 genes) also responded to the fasting-to-feeding transition (light green) (Fig. 3e). Strikingly, in the C1 and C2 clusters, hepatic gene expression in fasted Rab4b^HepKO^ mice was intermediate between that of fasted and fed Rab4b^fl/fl^ mice. Specifically, 77% (326 out of 423) of the genes upregulated in the liver of fasted Rab4b^HepKO^ animals (vs fasted Rab4b^fl/fl^ mice) were also induced by feeding in Rab4b^fl/fl^ mice (Fig. 3f). Conversely, 70.3% (216 out of 307) of the genes downregulated in the liver of fasted Rab4b^HepKO^ mice (vs fasted Rab4b^fl/fl^ mice) were likewise repressed by feeding in the liver of Rab4b^fl/fl^ mice. By performing ingenuity upstream regulator analysis, we found that some transcription factors, such as *Srebf2*, *Mlxipl*, and *Pparα*, were regulated consistently with the fasting state of the mice^26^, while others, including *E2f1* and *Nfe2l2*, were more coherent with a fed state^27,28^ (Fig. 3g). These results support that the transcriptional regulation in the liver of Rab4b depleted mice is disturbed.

Taken together, these results show that the absence of Rab4b in hepatocytes has profound effects on the liver transcriptome in the fasting state, reprograming the metabolic transcriptome towards a feeding-like state.

### Rab4b depletion in hepatocytes induces fasting hyperglycemia

**Fig. 4:**
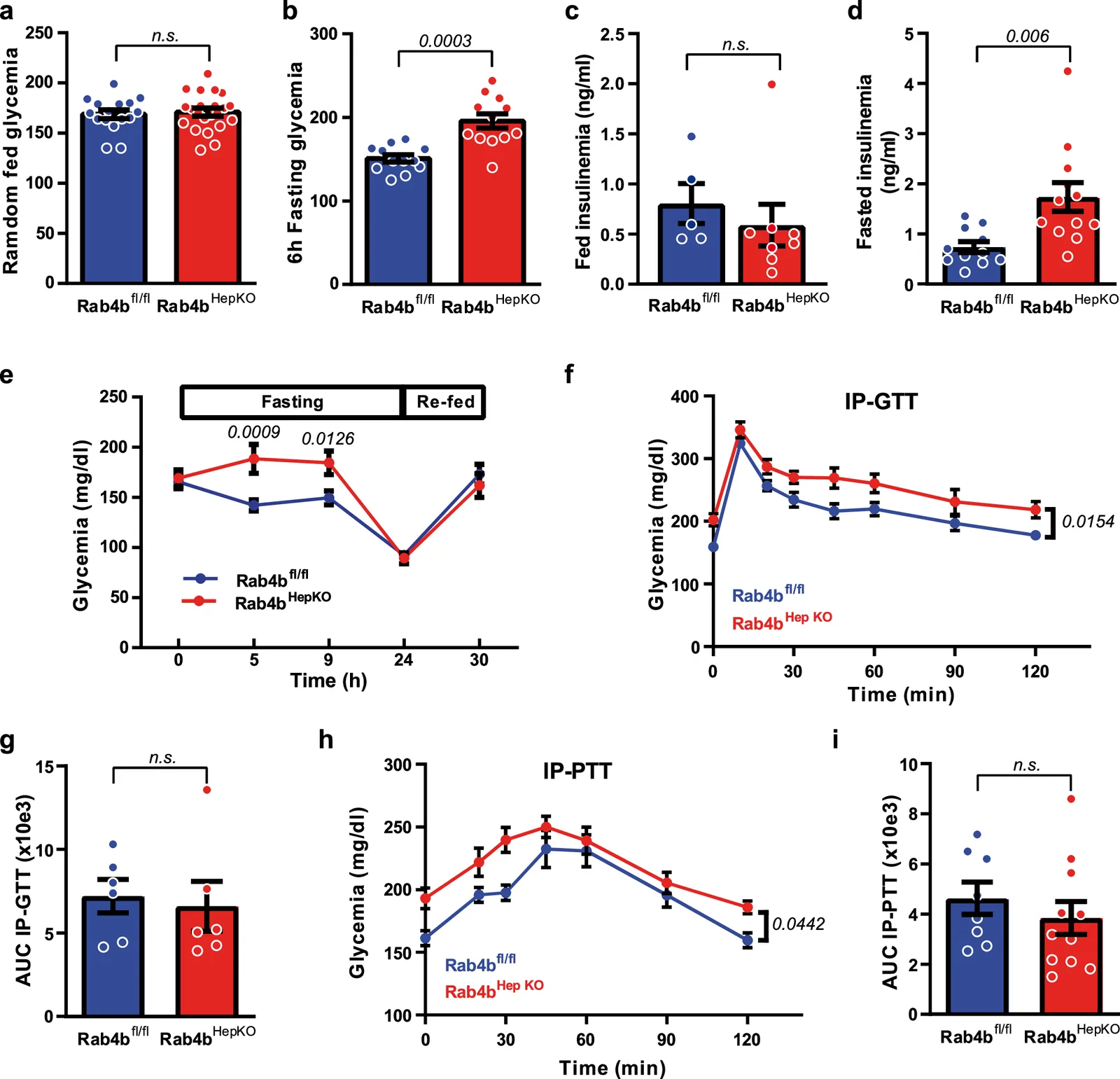
Rab4b depletion in mice hepatocyte in vivo leads to hyperglycemia upon fasting. **a** Glycemia (mg/dl) in random-fed Rab4b^fl/fl^ and Rab4b^HepKO^ mice (*n* = 16 Rab4b^fl/fl^ mice and 22 Rab4b^HepKO^ mice). **b** Glycemia (mg/dl) in 6 h-fasted Rab4b^fl/fl^ and Rab4b^HepKO^ mice (*n* = 12/genotype). **c** Insulinemia in random-fed Rab4b^fl/fl^ and Rab4b^HepKO^ mice (*n* = 5 Rab4b^fl/fl^ mice and 8 Rab4b^HepKO^ mice). **d** Insulinemia in 6 h-fasted Rab4b^fl/fl^ and Rab4b^HepKO^ mice (*n* = 11 Rab4b^fl/fl^ mice and 12 Rab4b^HepKO^ mice). **e** Change in blood glucose levels in Rab4b^fl/fl^ and Rab4b^HepKO^ mice upon 5, 9 and 24 h of fasting and 6 h of refeeding (*n *= 6/genotype). **f** Intraperitoneal (IP) glucose tolerance test (GTT) in Rab4b^fl/fl^ and Rab4b^HepKO^ mice (*n* = 6/genotype). **g** Area under the curves from the IP-GTT presented in (**f**). (*n* = 6/genotype). **h** Intraperitoneal (IP) pyruvate tolerance test (PTT) in Rab4b^fl/fl^ and Rab4b^HepKO^ mice (*n* = 8 Rab4b^fl/fl^ mice and 11 Rab4b^HepKO^ mice). **i** Area under the curves from the IP-PTT presented in (**h**) (*n* = 8 Rab4b^fl/fl^ mice and 11 Rab4b^HepKO^ mice). Error bars indicate means ± s.e.m. Unpaired two-sided t-test was used for panels (**a**, **i**), unpaired two-sided t-test with Welch’s correction for panels (**b**, **d**), unpaired two-sided Mann-Whitney test for panels (**c**, **g**), and two-way ANOVA for panels (**e**, **f**, **h**). Source data are provided as a Source Data file.

Transcriptome reprogramming from fasting to feeding in vivo is primarily regulated by glucose and insulin. To determine whether glycemia and insulinemia were altered in Rab4b^HepKO^ mice, we measured their levels under various nutritional states. After a 6-h fast, Rab4b^HepKO^ mice exhibited a 30% increase in blood glucose concentration compared to Rab4b^fl/fl^ mice, whereas glycemia under random-fed conditions remained similar between both genotypes (Fig. 4a, b). In line with these observations, fasting insulin levels were approximately twice as high in Rab4b^HepKO^ mice compared to Rab4b^fl/fl^ mice, whereas insulin levels under fed conditions did not differ significantly (Fig. 4c, d). We further measured the variation of glycemia across different fasting durations (5 h, 9 h, 24 h) and after a 6-h refeeding period (Fig. 4e). A 5-h fast caused a slight decrease in blood glucose levels in Rab4b^fl/fl^ mice but led to an increase in Rab4b^HepKO^ mice. This difference in glycemia persisted up to at least 9 h of fasting. After a prolonged 24-h fast, both genotypes exhibited a similar marked decrease in blood glucose concentration, although the kinetics of this decline may differ between them. Subsequently, refeeding fully restored glycemia, with no significant differences observed between genotypes. Apart from this difference in glycemia after short-term fasting, no change was observed in circulating levels of non-esterified fatty acids, triglycerides, total cholesterol, high-density cholesterol, low-density cholesterol, and corticosterone (Supplementary Fig. 7a, b). Consistent with the increase in blood glucose and insulin observed in fasted Rab4b^HepKO^ mice, we found a reduction in circulating glucagon (Supplementary Fig. 7c).

Given that Rab4b^HepKO^ mice presented a fasting hyperglycemia, we next assessed their glucose metabolism using glucose and pyruvate tolerance tests. Glycemic excursions during both tests were similar between the two genotypes, with the only difference being elevated basal glycemia in Rab4b^HepKO^ mice after a 6-h fast (Fig. 4f–i). Consistently, both the Quantitative Insulin Sensitivity Check Index (QUICKI)^29^ and Triglyceride-Glucose (TyG) index^30^ were comparable between genotypes (Supplementary Fig. 7d, e), suggesting that the observed fasting hyperglycemia in Rab4b^HepKO^ mice was unlikely to result from insulin resistance.

Taking together, these results indicate that the hepatocyte-specific depletion of Rab4b is sufficient to induce transient fasting hyperglycemia, which may contribute to the observed transcriptomic remodeling in the liver during physiologically short-term fasting in Rab4b^HepKO^ mice.

### Rab4b depletion in hepatocytes favors glucose production via glycogen breakdown

**Fig. 5:**
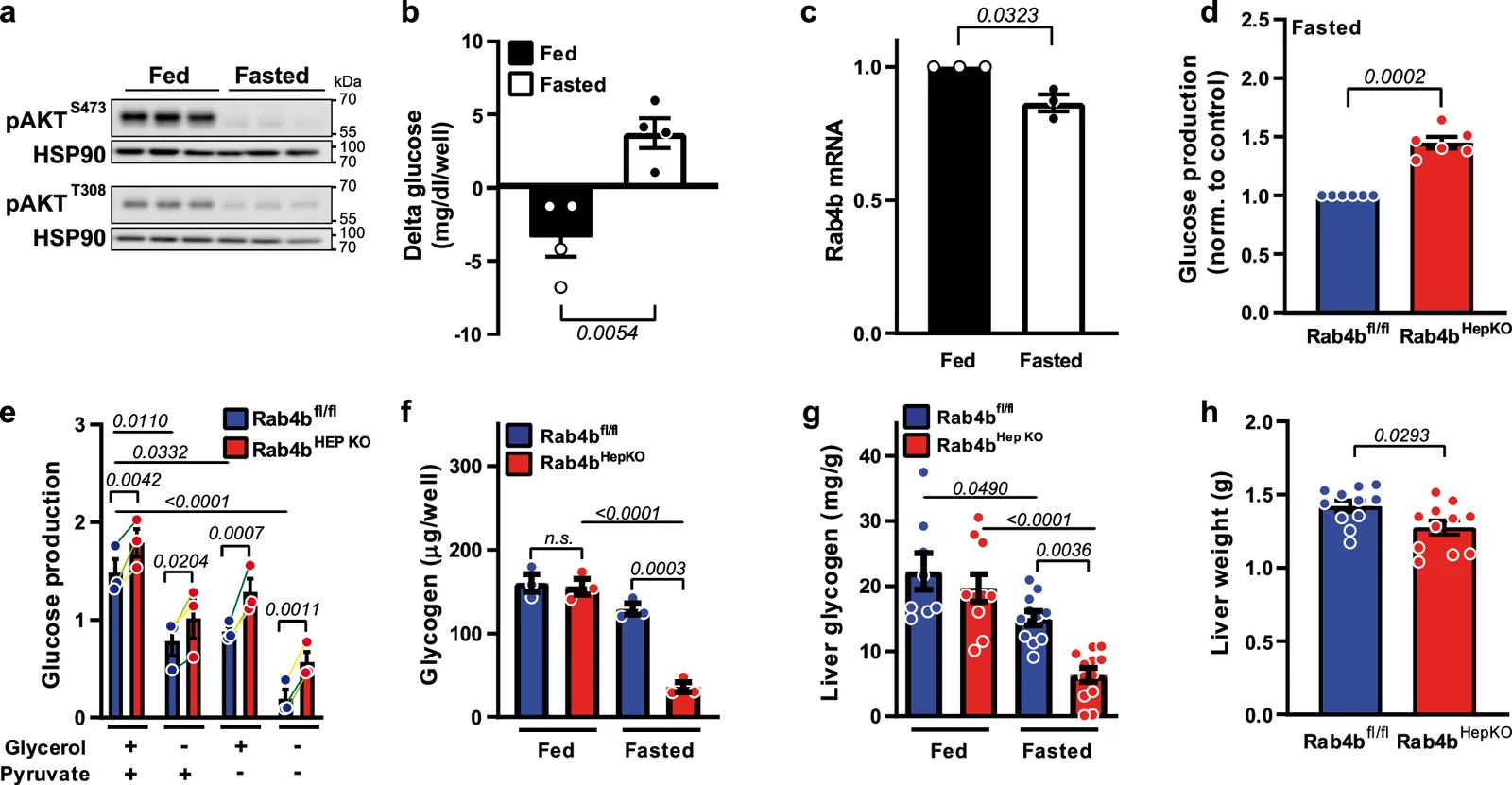
Rab4b depleted hepatocytes produce more glucose from glycogen upon fasting. **a** Representative Western Blot of Akt phosphorylation (on serine 473 and threonine 308) with Hsp90 as loading control in control primary mouse hepatocytes treated for 4 h with feeding or fasting culture media (*n* = 3/conditions). **b** Delta of the glucose level in culture media of control primary hepatocyte after 4 h of treatment with either feeding or fasting media versus the glucose level at time 0 h. This delta provides the amount of glucose produced ( > 0) or consumed ( < 0) by the primary hepatocytes during the 4 h incubation period. (*n* = 4/conditions). **c** *Rab4b* mRNA level in control primary mouse hepatocytes treated for 4 h with feeding or fasting culture media. (*n* = 3/genotype). **d** Glucose production (estimated as in (**b**)) in Rab4b^fl/fl^ and Rab4b^HepKO^ primary mouse hepatocytes treated for 4 h with fasting culture media. (*n* = 6/genotype). **e** Glucose production (estimated as in (**b**)) in Rab4b^fl/fl^ and Rab4b^HepKO^ primary mouse hepatocytes treated for 4 h with fasting culture media lacking or not glycerol, pyruvate or both. Biological replicates are represented with dark green, light green and yellow connecting lines (*n* = 3/conditions). **f** Glycogen level in Rab4b^fl/fl^ and Rab4b^HepKO^ primary mouse hepatocytes treated for 4 h with fasting or feeding media (*n* = 3/genotype). **g** Glycogen level in livers from Rab4b^fl/fl^ and Rab4b^HepKO^ mice either fed (*n* = 8 Rab4b^fl/fl^ and 10 Rab4b^HepKO^ mice) or fasted (*n* = 11 Rab4b^fl/fl^ and 12 Rab4b^HepKO^ mice). **h** Liver weight (**g**) in 6 h-fasted Rab4b^fl/fl^ and Rab4b^HepKO^ mice (*n* = 11/genotype). Error bars indicate means ± s.e.m. Unpaired two-sided t-test was used for panels (**b**, **h**), unpaired two-sided t-test with Welch’s correction for panels (**c**, **d**), two-way mixed-effects ANOVA with Sidak multiple-comparisons for panels (**e**), and two-way ANOVA with Sidak multiple-comparisons for panels (**f**, **g**). Source data are provided as a Source Data file.

To determine whether fasting hyperglycemia in Rab4b^HepKO^ mice originated from a hepatocyte-autonomous mechanism, we established primary mouse hepatocyte culture conditions that mimic the in vivo glucose metabolic responses in fed and fasted states. The fed culture conditions activated insulin signaling as evidenced by Akt phosphorylation (Fig. 5a) and promoted glucose consumption by hepatocytes (Fig. 5b). Conversely, the fasted culture conditions enhanced glucose production by hepatocytes (Fig. 5b). Importantly, upon these culture conditions, we observed a decrease in *Rab4b* mRNA expression in fasting (Fig. 5c). These culture conditions were therefore well-suited to investigate the cell-autonomous effects of Rab4b depletion on hepatocyte glucose handling. Strikingly, hepatocytes isolated from Rab4b^HepKO^ mice produced 1.5-times more glucose under fasting conditions compared to control hepatocytes (Fig. 5d). Overexpression of GFP-Rab4b, achieved by lentiviral infection in more than 80% of primary mouse hepatocytes (Supplementary Fig. 8a, b), did not, however, alter glucose production (Supplementary Fig. 8c). Underscoring the translational relevance of our findings, the RNAi-based silencing of *RAB4B* in human primary hepatocytes also led to a similar increase in glucose production (Supplementary Fig. 9a, b). This increase in glucose production by hepatocyte lacking Rab4b may contribute to the hyperglycemia observed in fasted Rab4b^HepKO^ mice.

In hepatocyte, fasting-induced glucose production can arise from either gluconeogenesis or glycogen breakdown. To assess whether gluconeogenesis was induced in Rab4b^HepKO^ hepatocytes, we removed glycerol and pyruvate, two essential fuels for gluconeogenesis, from the fasted culture media. Omitting either glycerol or pyruvate reduced glucose production from cultured Rab4b^fl/fl^ hepatocytes by ~50% (Fig. 5e). Removing both substrates decreased glucose production by ~86%, indicating that the remaining glucose likely derives from glycogen breakdown. However, under the same conditions, Rab4b^HepKO^ hepatocytes still exhibited higher glucose production than Rab4b^fl/fl^ hepatocytes, suggesting that gluconeogenesis is not the primary driver of the increased glucose output. This is consistent with the similar glycemic excursion between the two genotypes during the pyruvate tolerance test (Fig. 4h, i).

We next analyzed glycogen content in primary hepatocytes from both genotypes under fed and fasted culture conditions. In the fed conditions, glycogen levels were similar between genotypes both in mouse and human, indicating that glycogen storage upon feeding was not altered by the lack of RAB4B in hepatocytes (Fig. 5f and Supplementary Fig. 9c). In contrast, under fasting conditions, glycogen depletion was more pronounced in mouse and human hepatocytes lacking RAB4B, indicating that glycogen breakdown is more active. In the liver, in vivo glycogen content was similar between Rab4b^fl/fl^ and Rab4b^HepKO^ mice in the fed state. In contrast, 6 h of fasting induced a more pronounced reduction in glycogen content in the liver of Rab4b^HepKO^ mice (Fig. 5g). In line with this decrease, we also observed a liver weight reduction in Rab4b^HepKO^ mice (Fig. 5h), consistent with the fact that liver glycogen can account for up to 2.5% of total liver mass^31^.

Overall, these results demonstrate that Rab4b depletion in hepatocytes strongly enhances glycogen breakdown during short-term fasting, which may underline the observed fasting hyperglycemia of the Rab4b^HepKO^ mice.

### Depletion of Rab4b in hepatocytes induces glycophagy

In hepatocytes, glycogen breakdown occurs via two mechanisms, glycogenolysis and glycophagy^2,5^. Glycogenolysis is regulated by glycogen phosphorylase, whose activity is enhanced by phosphorylation^2^. We assessed glycogen phosphorylase activity in the livers of Rab4b^fl/fl^ and Rab4b^HepKO^ mice following a 6-h fast, a time point at which Rab4b^HepKO^ mice displayed hyperglycemia. No significant differences in glycogen phosphorylase activity were observed between the two genotypes (Supplementary Fig. 10a). In line with this observation, phosphorylation of glycogen phosphorylase was also comparable and, as expected, increased upon fasting in both groups (Supplementary Fig. 10b, c). To further assess the contribution of glycogenolysis, we pharmacologically inhibited glycogen phosphorylase using DAB^32^. This inhibition reduced glucose production in Rab4b^fl/fl^ hepatocytes cultured under fasting conditions but had no significant effect on Rab4b^HepKO^ hepatocytes (Supplementary Fig. 10d). These findings indicate that glycogenolysis is not the main driver of the elevated glucose production observed in Rab4b^HepKO^ hepatocytes.

**Fig. 6:**
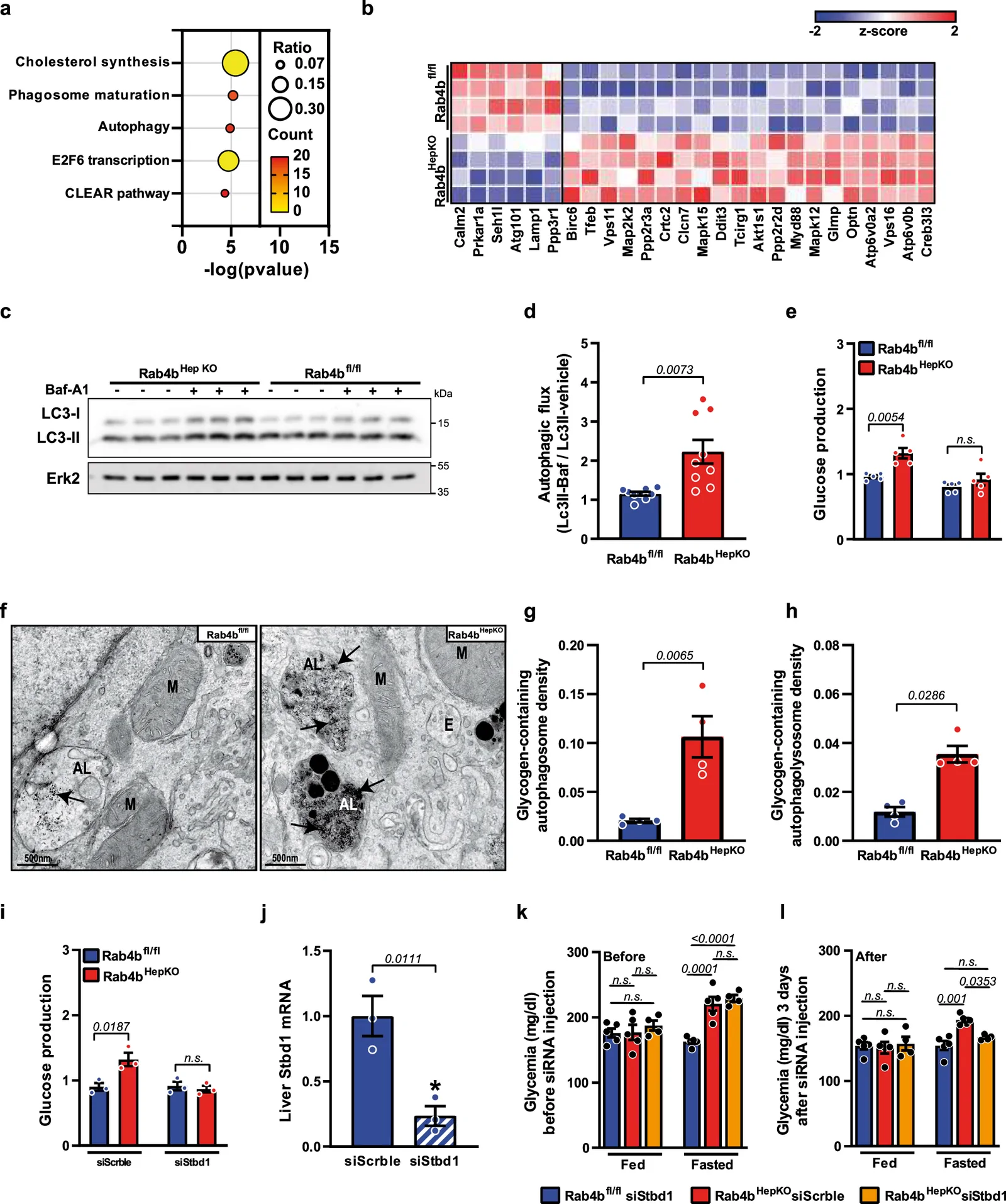
Depletion of Rab4b in hepatocytes induces glycophagy. **a** Ingenuity Pathway Analysis canonical pathway enrichment of differentially expressed genes between fasting Rab4b^fl/fl^ and Rab4b^HepKO^ liver. **b** Heatmap of autophagy and CLEAR pathway genes in fasted Rab4b^fl/fl^ and Rab4b^HepKO^ liver. **c** Representative LC3 westernblot from fasted Rab4b^fl/fl^ and Rab4b^HepKO^ primary mouse hepatocytes treated or not with Bafilomycin-A1. Erk2 is used as loading control. (*n* = 9/conditions). **d** Quantification of the autophagic flux in fasted Rab4b^fl/fl^ and Rab4b^HepKO^ primary mouse hepatocytes from immunoblots presented in (c) and in Supplementary Fig. 11. (*n* = 9/conditions). **e** Glucose production in Rab4b^fl/fl^ and Rab4b^HepKO^ primary mouse hepatocytes treated for 4 h with fasting culture media complemented with or without Bafilomycin-A1 (*n* = 5/conditions).** f** Representative electron microscopy images from Rab4b^fl/fl^ and Rab4b^HepKO^ primary mouse hepatocyte treated for 4 h with fasting culture media (*n* = 4/conditions). Mitochondria (M), autolysosome (AL) and endosomes (E) are labeled, and some glycogen granules are marked with arrows. **g**, **h** Density (number / µm^2^) of autophagosome (**g**) and autolysosome (**h**) containing glycogen granules (*n* = 4/conditions). **i** Glucose production in Rab4b^fl/fl^ and Rab4b^HepKO^ primary mouse hepatocytes treated for 4 h with fasting culture media following in vitro silencing with Scramble (control), or *Stbd1* siRNA (*n* = 3/conditions). **j** *Stbd1* mRNA levels in liver from control mice treated with Scramble siRNA (control) or *Stbd1* siRNA (*n* = 3/conditions). **k–l** Fed and fasted glycemia in Stbd1 siRNA-treated Rab4b^fl/fl^ mice (blue bars - *n* = 5 mice) or Rab4b^HepKO^ mice (orange bars - *n* = 4 mice) and in Rab4b^HepKO^ mice treated with Scramble siRNA (red bars - *n* = 5 mice), before (**k**) and 3 days after siRNA injection (**l**). Error bars indicate means ± s.e.m. Unpaired two-sided t-test with Welch’s correction was used for panel (d), two-way ANOVA with Sidak multiple-comparisons for panels (**e**, **i**, **k**, **l**), unpaired two-sided t-test for panel (**g**, **j**), and unpaired two-sided Mann-Whitney test for panel (**h**). Source data are provided as a Source Data file.

Glycophagy is a glycogen-selective autophagy process^6^. Interestingly, canonical pathway analysis of the genes differentially expressed in the liver of fasted Rab4b^HepKO^ mice compared to fasted Rab4b^fl/fl^ mice highlighted lysosome biogenesis (CLEAR pathway) and autophagy (Fig. 6a). Most of the genes involved in these two pathways are induced in the liver of fasted Rab4b^HepKO^ mice (Fig. 6b), suggesting a coordinated activation of lysosome biogenesis and autophagy. To assess this possibility, we measured LC3 levels by Western blot as a readout for autophagic flux in hepatocytes under fasting conditions. LC3-II levels were lower in Rab4b^HepKO^ hepatocytes than in Rab4b^fl/fl^ hepatocytes (Fig. 6c), which could reflect either reduced autophagy initiation or increased autophagic flux. To distinguish between these possibilities, we treated cells with Bafilomycin A1 (Baf-A1), an inhibitor of autophagosome-lysosome fusion that blocks autophagic degradation. In Rab4b^fl/fl^ hepatocytes, Baf-A1 treatment caused only a modest increase in LC3-II levels, indicating a relatively low basal autophagy flux (Fig. 6c and Supplementary Fig. 11). In contrast, Rab4b^HepKO^ hepatocytes exhibited a substantial accumulation of LC3-II following Baf-A1 treatment, revealing a significantly enhanced autophagic flux (Fig. 6c, d). To determine whether this increase in autophagy contributes to the elevated glucose production observed in Rab4b^HepKO^ hepatocytes, we measured glucose output in primary hepatocytes cultured under fasting when autophagy is blocked. Strikingly, Baf-A1 treatment normalized glucose production in Rab4b^HepKO^ hepatocytes to levels comparable to those of Rab4b^fl/fl^ hepatocytes (Fig. 6e). These results reveal that Rab4b depletion in hepatocytes promotes glucose production through an autophagy-dependent process.

To determine whether Rab4b depletion favors glycophagy, we performed transmission electron microscopy to quantify the number of autophagy-related organelles that contain glycogen. In primary hepatocytes cultured under fasting conditions, glycogen granules (black dots, indicated by arrows) were detected within autophagic compartments in both genotypes (Fig. 6f). However, the number of autophagosomes and autolysosomes containing glycogen was significantly higher in Rab4b^HepKO^ hepatocytes compared to controls (Fig. 6f–h). We then determined the impact of glycophagy disruption on glucose output in fasted conditions, by using siRNA-mediated knockdown of *Stbd1* (Supplementary Fig. 12a), a selective autophagy receptor for glycogen^33^. Notably, *Stbd1* silencing in Rab4b^HepKO^ hepatocytes restores glucose production to control levels (Fig. 6i).

To assess the contribution of glycophagy to the fasting-induced hyperglycemia observed in Rab4b^HepKO^ mice, we performed siRNA-mediated silencing of *Stbd1* in the liver of Rab4b^HepKO^ mice and Rab4b^fl/fl^ mice. This intervention resulted in an ~70% reduction in hepatic Stbd1 mRNA and protein levels compared to control mice treated with scrambled siRNA (Fig. 6j and Supplementary Fig. 12b,c). Prior to siRNA injection, Rab4b^HepKO^ mice were randomized into two groups with comparable blood glucose levels under both fed and fasted state. One group was treated with Scramble siRNA and the other with Stbd1 siRNA. In line with our previous findings, Rab4b^HepKO^ mice exhibited similar glycemia to Rab4b^fl/fl^ mice in the fed state but displayed significantly higher glycemia after 6 h of fasting before siRNA administration (Fig. 6k). Importantly, three days after siRNA administration, fasting hyperglycemia in Rab4b^HepKO^ mice was effectively reversed, with blood glucose levels restored to values comparable to those of Rab4b^fl/fl^ mice, whereas Rab4b^HepKO^ mice treated with scrambled siRNA continued to exhibit elevated fasting glycemia (Fig. 6l).

These results demonstrate that the loss of Rab4b in hepatocytes leads to fasting hyperglycemia by enhancing liver glucose production through increased glycophagy.

### Depletion of Rab4b in hepatocytes favors autophagy through transferrin receptor trafficking

**Fig. 7:**
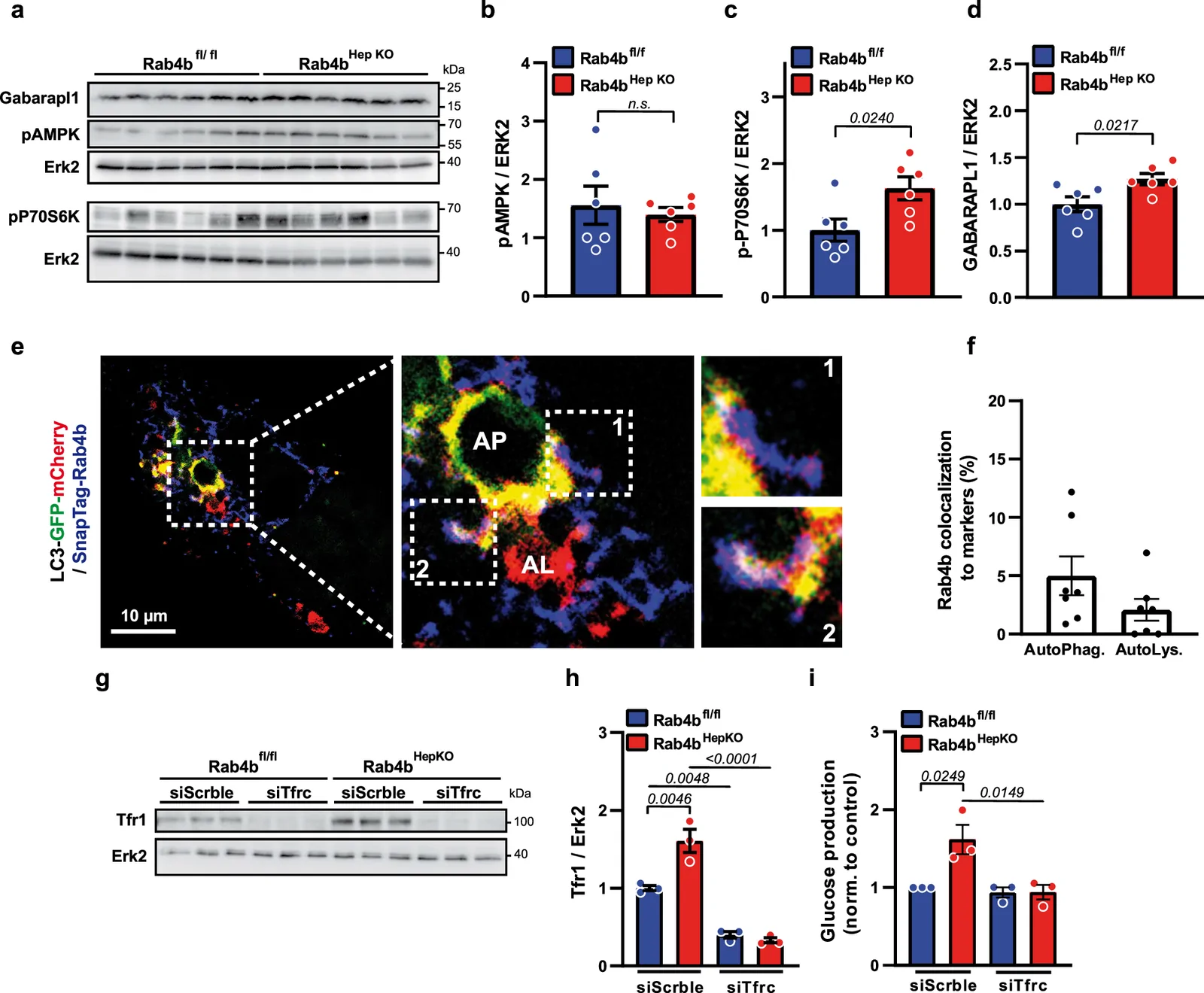
The increase in glucose production in Rab4b-depleted hepatocytes requires transferrin receptor. **a** Western Blot of Gabarapl1, pAMPK^Thr172^ and p-p70S6K^Thr389^ in liver lysates from fasted Rab4b^fl/fl^ and Rab4b^HepKO^ mice (*n* = 6/genotypes). Erk2 is used as loading control. **b** Quantification of pAMPK from the immunoblot presented in (**a**). **c** Quantification of p-p70S6K from the immunoblot presented in (**a**). **d** Quantification of Gabarapl1 from the immunoblot presented in (**a**). **e** Representative SRRF image of primary hepatocytes isolated from Rab4b-SnapTag mice, transfected with the LC3-GFP-mCherry construct and immunolabelled with and anti SnapTag antibody, allowing detection of Rab4b (blue), autophagosomes (AP–in yellow) and autolysosomes (AL – in red). **f** Quantification of the percentage of Rab4b colocalized with autophagosomes and autolysosomes (*n* = 7/conditions). **g** Western Blot of the transferrin receptor (Tfr1/CD71) in primary mouse hepatocytes isolated from the liver of Rab4b^fl/fl^ and Rab4b^HepKO^ mice and treated with Scramble siRNA (control) or anti Tfrc siRNA (*n* = 3/conditions). Erk2 is used as loading control. **h** Quantification of Tfr1 from the immunoblot presented in (**a**). **i** Glucose production in Rab4b^fl/fl^ and Rab4b^HepKO^ primary mouse hepatocytes treated for 4 h with fasting culture media following in vitro silencing with Scramble (control), or *Tfrc* siRNA (*n* = 3/conditions). Error bars indicate means ± s.e.m. Unpaired two-sided t-test with Welch’s correction was used for panel (**b**), unpaired two-sided t-test for panels (**c**, **d**), and two-way ANOVA with Sidak multiple-comparisons for panels (**h**, **i**). Source data are provided as a Source Data file.

Glycophagy depends on both the induction of autophagy, which regulates phagophore formation and autophagic flux, and the selective recognition of glycogen for its recruitment on into the autophagosome. To determine how the depletion of Rab4b induces autophagy, we first investigated AMPK and mTOR signaling. Indeed, activation of AMPK (through phosphorylation of its α‑subunit at Thr172) promotes autophagy, whereas activation of mTOR suppresses it^34^. We found that the phosphorylation of AMPK upon fasting was not significantly changed in the liver of Rab4b^HepKO^ liver compared to Rab4b^fl/fl^ mice (Fig. 7a, b). In addition, the phosphorylation of p70S6K, a downstream target of mTOR, was increased in fasted Rab4b^HepKO^ livers (Fig. 7a, c), likely in response to elevated insulin levels. Afterward, we analyzed the expression of Gabarapl1, the ATG8 family member that facilitates glycophagy^6^. Interestingly, Gabarapl1 protein level was modestly increased by 1.2-fold in fasted Rab4b^HepKO^ mice compared to fasted Rab4b^fl/fl^ mice (Fig. 7a, d). Although, this increase could participate in the recruitment of the glycogen on the phagophore, Atg8 family members are not sufficient to drive autophagy initiation^35^. Therefore, none of these pathways could explain the increase in autophagic flux we observed in Rab4b^HepKO^ hepatocytes.

Next, we investigated the relationship between Rab4b and autophagic structures by analyzing its localization relative to autophagosomes and autolysosomes. We used LC3-GFP-mCherry to differentially label autophagic structures. LC3-GFP-mCherry localizes to autophagosomes, where both GFP and mCherry fluoresce, resulting in an overlapping yellow signal. In autolysosomes, GFP fluorescence is quenched in the acidic environment, leading to red fluorescence. This construct was transduced into primary mouse hepatocytes isolated from *SnapTag-Rab4b* knock-in mice, expressing at endogenous levels a tagged version of Rab4b, labeled in blue. Using Super-Resolution Radial Fluctuations (SRRF) microscopy (Fig. 7e), we observed that Rab4b is barely detectable in autophagic structures, in accordance with its early and recycling endosome localization. However, a small fraction, around 10%, is nevertheless associated with these structures as evidenced by purple (autolysosomes) and white (autophagosomes) color (see insets) (Fig. 7f). Therefore, we cannot exclude the possibility that Rab4b-containing structures may transiently interact with autophagic vacuoles.

Recently, the transferrin receptor, whose endocytic trafficking is largely regulated by Rab4b^17^, as confirmed here (Fig. 2g–i), was found to be pivotal for autophagy induction and autophagosome closure^36^. Hence, we tested whether the transferrin receptor is involved in the increase in glycophagy observed in Rab4b-depleted hepatocytes. Strikingly, silencing the transferrin receptor reversed the elevated glucose production seen in Rab4b^HepKO^ hepatocytes (Fig. 7g–i).

Together, these results demonstrate that the loss of Rab4b in hepatocytes promotes autophagy, and therefore glycophagy, by increasing intracellular transferrin receptor levels.

## Discussion

Here, we show that during fasting, glycophagy may contribute to hepatic glycogen breakdown by repressing the expression of the endosomal small GTPase Rab4b. Indeed, we found that Rab4b expression is reduced under fasting conditions, and that liver-specific deletion of *Rab4b* leads to a transient increase in blood glucose during fasting, accompanied by decreased hepatic glycogen stores. Notably, this effect is not driven by conventional glycogenolysis, but rather by Stbd1-dependent glycophagy.

Our results further support the concept that endocytic trafficking regulates liver metabolism in vivo^9,10^. In lipid and cholesterol metabolism, both endolysosomal degradation and recycling pathways have been implicated^37^. In contrast, for glucose metabolism, only the degradation pathway has been linked to hepatic glucose production, while the role of recycling remains largely unexplored. Prior studies show a strong link between endolysosomal degradation and G6Pase-dependent gluconeogenesis. As example, caveolin-1 expression is essential for G6Pase-dependent glucose production^38^. In addition, loss of endosome and lysosome through Rab5 silencing represses *G6pc* expression^11,12^, whereas impaired early-to-late endosome maturation through *Vps37a* knockdown increases *G6pc* expression^13^. Here, we show that altered endocytic recycling can induce fasting hyperglycemia via G6Pase-independent mechanism. Thus, endocytic recycling also regulates hepatic glucose production through distinct pathways.

Our in vivo and ex vivo data reveal glycophagy as a key pathway for hepatic glycogen breakdown that supports blood glucose during fasting. In Rab4b-deficient mice, increased hepatic glucose production results from enhanced glycophagy. This effect is reversed by *Stbd1* silencing, the adapter linking glycogen to the phagophore for lysosomal degradation^33,39^. While well studied in the neonatal period^7^, when gluconeogenesis is not yet fully functional^40,41^, glycophagy’s role in adults liver has been largely overlooked. Adults are typically thought to rely solely on enzymatic glycogenolysis and gluconeogenesis to maintain fasting glucose, which may explain the limited focus on glycophagy. Hence, the physiological role of glycophagy in adult glucose regulation has remained unclear. In Pompe disease, where lysosomal glycogen accumulates instead of being degraded due to acid α-glucosidase (*GAA*) deficiency, the lack of reports on blood glucose dysregulation has led to the belief that glycophagy is dispensable for glucose homeostasis. However, in rodent, insulin, a key inhibitor of glycogen breakdown and gluconeogenesis, has also been shown to suppress liver glycophagy^42^. Consistently, adult mice lacking *Gaa* present a significant hypoglycemia during fasting^43^, suggesting that glycophagy plays a role in adult hepatic glucose production. Our findings further support the idea that glycophagy contributes to glucose homeostasis in adult mice and is dynamically regulated during the transition from feeding to fasting.

The chronological sequence of activation of gluconeogenesis, glycogenolysis, and glycophagy during fasting to produce glucose remains incompletely understood. Recent findings indicate that intracellular glycogen inhibits gluconeogenesis^44^, suggesting that gluconeogenesis depends on prior glycogen breakdown. Whereas glycogenolysis, driven by glycogen phosphorylase, is rapidly activated during the first h of fasting^45^, the timing of glycophagy activation remains unclear. Our results indicate that glycophagy can be triggered by Rab4b depletion. Although its complete depletion may not fully reflect the physiological ~50% reduction we observed during fasting, Rab proteins are highly sensitive to haploinsufficiency^46,47^, and studies in Drosophila show that partial loss often phenocopies complete loss^48^. This supports the possibility that glycophagy may be initiated after 6 h of fasting, when Rab4b expression physiologically declines. This timing is compatible with a role for glycophagy in clearing abnormal glycogen particles at the end of the glycogen phosphorylase activity, as recently proposed^49^.

Although both glycophagy and glycogenolysis generate glucose, they occur in different subcellular compartments: the lysosome and the endoplasmic reticulum, respectively. This suggests that they may fulfill different cellular metabolic functions. Indeed, glucose derived from glycophagy in lysosomes may affect nutrient-sensing pathways locally at the lysosomal membrane, where the opposing kinases mTORC1 and AMPK respond to energy status^50,51^. Both are sensitive to glycolytic intermediates^52,53^, and recent studies show that glycolytic enzymes can localize to sites of high energy demand^54–56^. Although the direct coupling of glycolysis to lysosomes remains unproven, this raises the possibility that glycophagy acts as a metabolic rheostat, regulating mTORC1 and AMPK activity to fine-tune the hepatocyte energy metabolism during physiological fasting, beyond merely supporting systemic glucose delivery.

We previously demonstrated that Rab4a and Rab4b have opposing effect on endosomal recycling. Rab4a favors direct trafficking from early endosomes to the plasma membrane, when Rab4b mediates trafficking to recycling endosomes^17^. Here, we show that Rab4b acts as a negative regulator of autophagy, whereas Rab4a has been involved in autophagosome formation^57^. These opposite roles highlight how cargo routing at early endosomes can critically influence autophagy regulation. Following sorting in early endosomes, cargo can be directed to recycling endosomes, which play a key role in autophagy. Beyond supplying membrane to phagophores^58^, recycling endosomes serve as platforms for the recruitment of essential autophagy machinery. Notably, Atg9 and Atg16l1, which are required for phagophore nucleation and expansion, depend on trafficking through this compartment^59–62^, with Atg16l1 recruitment being mediated by Rab11a^63^, a marker of recycling endosomes. Consistently, the overexpression of transferrin receptor has been shown to induce autophagy flux by promoting the recruitment of Wipi2, Atg16l1 and Atg5 on Rab11a-positive compartments^36^. Our results indicate that Rab4b drives this pathway by modulating transferrin receptor trafficking, thereby favoring glycogen degradation through autophagy.

In conclusion, taken together, our results uncover a mechanism whereby Rab4b acts as a brake on glycophagy. During physiological fasting, the decline in blood glucose levels leads to reduced Rab4b expression. This downregulation disrupts transferrin receptor trafficking, promoting its intracellular accumulation, which has recently been shown to enhance autophagic flux^36^. In the fasting context, favorable to glycogen recruitment to the phagophore, this increase in autophagic flux ultimately leads to the induction of glycophagy. Glucose production, through glycophagy, may thereby contribute to the maintenance of blood glucose homeostasis.

## Methods

### Mouse

#### Housing

Mice were housed under controlled temperature (23 °C) and humidity conditions in a 12 h light/dark cycle with ad libitum access to food and water. Housing was performed according to the animal care guidelines of the European Community Council (2010/63/EU). All animal experiments were approved by the local ethics committee and the French Ministry of Higher Education and Research (CIEPAL-Azur NCE/2018-529).

#### Rab4b^HepKO^ mice generation and breeding strategy

C57Bl6 Rab4b^fl/fl^ mice were obtained under pure background at the Mouse Clinical Institute (ICS, Illkirch, France)^20^. Rab4b^fl/fl^ were crossed with mice expressing the Cre recombinase under the Albumin promoter to obtain Alb-Cre^+/-^ Rab4b^fl/wt^ mice. Alb-Cre^+/-^ Rab4b^fl/wt^ mice were crossed with Rab4b^fl/fl^ mice to obtain Alb-Cre^+/-^ Rab4b^fl/fl^ mice. Afterwards, Alb-Cre^+/-^ Rab4b^fl/fl^ mice were crossed with Rab4b^fl/fl^ mice to generate mice depleted for Rab4b in hepatocytes (Rab4b^HepKO^ mice) and their littermate control (Rab4b^fl/fl^ mice). The Rab4b^HepKO^ mice are viable and born at expected Mendelian ratio.

#### Rab4b^SnapTag^ mice generation

To detect Rab4b, for which antibodies are barely efficient, we generated C57Bl6 mice knocked-in for SnapTag in the *Rab4b* gene at the Jackson Laboratory. This strategy produce a Rab4b protein tagged at its N-terminal part with a SnapTag protein, which is expressed at the endogenous levels and easily detectable by using antibodies. Mice colony was maintained as homozygous animals.

#### Mice genotyping

DNA was extracted from mouse tails. To detect *Rab4b* with flox sequences the following PCR reaction procedure: 3 min at 94 °C / 5 cycles of 1 min at 94 °C, 1 min at 62 °C, 1 min at 72 °C / 40 cycles of 10 sec at 94 °C, 30 sec at 62 °C, 1 min at 72 °C / 3 min at 72 °C was performed with the following primers: to 5’-TGG CAC TTC CAG CAG TGG GT-3’ and 5’-TTC CCC TGC CTC TTC TGC CC-3’. To detect the Cre-recombinase simultaneously with an internal control the following PCR reaction procedure: 30 sec at 94 °C / 30 cycles of 30 sec at 95 °C, 30 sec at 60 °C, 1 min at 72 °C / 5 min at 72 °C was performed with the following primers: 5’-GCG GTC TGG CAG TAA AAA CTA TC-3’, 5’-GTG AAA CAG CAT TGC TGT CAC TT-3’, 5’-CTA GGC CAC AGA ATT GAA AGA TCT-3’ and 5’-GTA GGT GGA AAT TCT AGC ATC ATC C-3’. To detect the SnapTag the following PCR reaction procedure: 3 min at 94 °C / 5 cycles of 1 min at 94 °C, 1 min at 62 °C, 1 min at 72 °C / 28 cycles of 10 sec at 94 °C, 30 sec at 62 °C, 1 min at 72 °C / 2 min at 72 °C was performed with the following primers: to 5’- TGG ACA AAG ACT GCG AAA TG-3’ and 5’- CCA GCA GCC ACT CTT TCA C-3’.

#### STAM mice

Mice were obtained from the SMC Laboratories. C57BL/6 J mice (14-day-pregnant female, 6 weeks of age male) have been obtained from Japan SLC, Inc. (Japan). The animals have been maintained in a SPF facility under controlled conditions of temperature (23 ± 3 °C), humidity (50 ± 20%), lighting (12-h artificial light and dark cycles; light from 8 am to 8 pm) and air exchange. MASH was induced in male mice by a single subcutaneous injection of 200 µg streptozotocin (STZ, Sigma-Aldrich, USA) solution 2 days after birth and feeding with high fat diet (HFD, 57 kcal% fat, Cat# HFD32, CLEA Japan, Inc., Japan) after 4 weeks of age. The animals have been sacrificed at 24 weeks of age by exsanguination through direct cardiac puncture under isoflurane anesthesia (Pfizer lnc.).

#### Fasting/feeding conditions

20-week-old male mice were either sacrificed at 9 am with free access to food (considered as feeding status) or fasted at 9 am for the time indicated in the figure until their sacrifice. At sacrifice, serum and organs were harvested, snap frozen or fixed (4% paraformaldehyde or 2.5% glutaraldehyde) for further experiments.

#### Invivofectamin-based RNAi silencing

Ambion in vivo pre-designed siRNAs directed against a Scramble sequence as control (siScrble; Ambion 4457289; Sequence: UAACGACGCGACGACGUAATT – UUACGUCGUCGCGUCGUUATT) and Stbd1 (siStbd1; Ambion 4457308; Sequence: GGAAGUUACUCGUUGGGAATT –UUCCCAACGAGUAACUUCCTT) were complexed with Invivofectamin 3.0 reagent (Thermofisher) for 30 min at 50 °C, diluted and intravenously injected in 20 week-old male mice following customer protocol.

#### Metabolic exploration

Male mice were fed normal chow diet (SAFE, #A04) from 3-4 weeks of age. Intraperitoneal glucose tolerance tests (IP-GTT; 0.8 g/kg body weight) and intraperitoneal pyruvate tolerance tests (IP-PTT; 2 g/kg body weight) were conducted in 16-week-old mice following a 6-h fast. Glucose level was measured in tail vein blood using a glucometer. Serum insulin was measured using an HTRF-based assay (CisBio, Perkin Elmer). Serum NEFAs, triglycerides (TG), total cholesterol (TC), LDL, HDL and ALAT were measured using automated biochemical assays (Mindray BS240 pro, C3M). QUICK Index^29^ was calculated as following: Quick index = $1/(\log ({Fasting\; insulin\; \mu U}/{ml})+\log ({Fasting\; glucose\; mg}/{dL}))$. Tryglyceride-Glucose index (TyG index)^30^ was calculated as following: TyG index = ${{{\rm{Ln}}}}({Fasting\; glucose\; mg}/{dL\; x\; Fasting\; Tryglycerides\; mg}/{dL})/2$.

#### Measurement of glucocorticoid levels

Corticosterone was quantified by LC‑MS/MS^64^. After spiking 100 µL of calibrator, QC, or serum with 10 µL of internal standard (LC‑MS/MS steroid TDM kit, Chromsystems), samples underwent protein precipitation with methanolic zinc sulfate and phospholipid removal by Oasis HLB prime SPE (Waters). A 10 µL extract was injected into a Xevo TQ‑S micro mass spectrometer coupled to an Acquity UHPLC system (Waters). Separation was performed on an ACQUITY Premier BEH C18 column using a water/methanol gradient with 2 mM sodium acetate. Retention time was 4.8 min, and detection used positive ESI with two MRM transitions (m/z 347.24 → 121/329). Interassay CVs were 5.0–6.3%, with LOQs of 0.1 ng/mL.

### Primary hepatocyte

#### Isolation and culture

For primary mouse hepatocytes, Rab4b^HepKO^ or Rab4b^fl/fl^ male mice were anaesthetised with an intraperitoneal injection of 100 mg/kg Ketamine and 10 mg/kg Xylasine. Mouse livers were first perfused via the vena cava with HBSS buffer (ThermoFisher #14175053) containing 10 mM Hepes and 0.5 mM EGTA for 6 minutes. Next, livers were perfused with HBSS buffer containing 10 mM Hepes, 0.4 mM CaCl2 and 120 U/mL Collagenase (Sigma #C5138) for 10 minutes. Livers were harvested and dissociated in seeding medium, filtered through 100 µm filters and the cells were centrifuged at 50 g for 5 min. Living cells were obtained in the pellet following a centrifugation in Percoll media, and resuspended in William’s media complemented with 10% Fetal Calf Serum, 2 mM L-glutamine, 130 nM insulin and 1% penicillin/streptomycin. Cells seeded at 400,000 cells/mL were cultured at 37 °C and 5% CO_2_ in a humid chamber.

For primary human hepatocytes, cryopreserved primary human hepatocytes (PHH) from a single donor (HUM182701, healthy Caucasian male donor of 31 years old, BMI 22) were obtained from Lonza (Basel, Switzerland). PHH were thawed at 37 °C for a minute, washed in Williams’ E medium without FBS, and seeded at 2×10⁵ cells per well on collagen-coated plates in Williams’ E medium supplemented with 10% FBS, 100 nM dexamethasone, and 1% penicillin/streptomycin^65^.

#### Fasting- and feeding-mimicking culture conditions

For primary mouse hepatocytes, to reset intracellular response to signaling, adhering cells were depleted for 16 h in William’s media complemented with 1 mg/ml Bovin Serum Albumin and 1% penicillin/streptomycin. Second, cells were exposed for 4 h with culture media mimicking either fasting or feeding conditions. The fasting media is composed of DMEM containing 1 mM glucose and supplemented with 1 mM pyruvate, 2.5 mM glycerol, 2 mM L-Glutamine, 10 µM Forskolin, 1 mg/ml Bovin Serum Albumin and 1% penicillin/streptomycin. The feeding media is composed of DMEM containing 11 mM glucose and supplemented with 100 nM Insulin, 1 mg/ml Bovin Serum Albumin and 1% penicillin/streptomycin.

For primary human hepatocytes, 3 days after silencing, PHH were pre-starved for 8 h in phenol-red–free DMEM containing 0.5% FBS, 5 mM glucose, and 4 mM glutamine. Cells were then washed twice with PBS and incubated for 12 h in glucose-free DMEM containing 4 mM glutamine, 2 mM sodium pyruvate, 20 mM sodium lactate, 100 nM dexamethasone, 1 µM forskolin (F6886, Millipore), and 10 nM glucagon (05-23-2700, Millipore)^13^.

#### Glucose production

For primary mouse hepatocytes, to estimate the amount of glucose produced, glucose dosage was performed in culture media at time 0 and 4 h with the Glucose GOD FS kit (Diasys # 1 2500 99 10 021). Glucose production = [glucose T4h] – [glucose T0].

For primary human hepatocytes, glucose released during 12 h into the medium was quantified using a colorimetric Glucose Assay Kit (ab65333, Abcam, Cambridge, UK) and normalized to total cellular protein content per well (5000116, DC BCA assay, Bio-Rad).

#### Pharmacological inhibitors

Glycogenolysis was inhibited by 400 nM 1,4-dideoxy-1,4-imino-D-arabinitol (DAB), a pharmacological inhibitor of glycogen phosphorylase^32^. Autophagic process, including glycophagy, was inhibited by 100 nM Bafilomycin-A1 (Baf), a pharmacological inhibitor of lysosomal V-ATPase pumps.

#### In vitro RNAi silencing

Cells were incubated for 6 h with OptiMEM medium containing Interferin (Polyplus #409-10) complexed with SMARTpool RNAi directed against either a non-existent sequences in the genome (siScrble; Dharmacon #D-001810-10-20; Sequences: UGGUUUACAUGUCGACUAA – UGGUUUACAUGUUGUGUGA – UGGUUUACAUGUUUUCUGA – UGGUUUACAUGUUUUCCUA), Stbd1 (siStbd1; Dharmacon, #L-056047-01-0005; Sequences: UCGAGAAAGCAACGGACAU – GCUGAGGAUAGGCGAGACA – CUUACAUACCAGCGAGUGA – GGUACAAGGUAGAGCGCCC), or Transferrin Receptor (siTfrc; Dharmacon, #L-055550-01-0005; Sequences: CACUAAGGGUGUACGUAAU – AUGAGGAACCAGACCGUUA – GGAUAUGGGUCUAAGUCUA – CGGCAAGUAGAUGGAGAUA). Complex formation was obtained following the manufacturer’s recommendations.

For primary human hepatocytes, 3 h after attachment, cells were transfected with siRNA directed against a scramble sequence as control (siScrble; Dharmacon #D-001810-10-05; Sequences: UGGUUUACAUGUCGACUA – UGGUUUACAUGUUGUGUGA – UGGUUUACAUGUUUUUGA – UGGUUUACAUGUUUUCCUA) or RAB4B (siRAB4B; Dharmacon # L2-008780-01-0005; Sequences: UCACCAGCCGGGAGACAUA – GGUGAUUGGCAGUGCAGGA – GCUAGUACCUGUUAUUUAU – AGCUAGACCCGGAGAGGAU), using Lipofectamine™ RNAiMAX Transfection Reagent (Invitrogen, #13778150) according to the manufacturer’s instructions and incubated for 8 h in Williams’ E medium. Subsequently, a second collagen layer was overlaid to establish a 3D collagen sandwich, and cells were maintained at 37 °C, 5% CO₂ in WE with 10% FBS and penicillin/streptomycin, with daily medium changes.

#### Plasmid transfection

Primary mouse hepatocytes isolated from SNAP-Tag-Rab4b expressing mice were incubated with a mixture of jetPEI-hepatocyte (Polyplus) and the chimeric Plasmids construct LC3-GFP-mCherry for 6 h following the manufacturer’s recommendations. One day after transfection, cells were fixed for 20 min with 4% PFA at room temperature. SNAP-Tag immunolabelling was performed using anti SNAP-Tag antibody (New England Biolabs, #P9310S, Rabbit, 1/1’00). High-speed time-lapse of 400 images were acquired at full speed on a Nikon spinning-disk microscope equipped with a 40x water immersion objective (C3M imaging facility, Nice). Super-Resolution Radial Fluctuations (SRRF) image analysis was performed^18^ using a ring radius of 0.5 and a magnification of 5.

#### Rab4b overexpression

Primary mouse hepatocytes were infected with lentivirus (VVTG Necker, Paris) expressing either GFP (control) or GFP-Rab4b with a multiplicity of infection of 10. Two days after the infection, glucose production upon fasting was tested as described above. Quantification of the number of cells overexpressing GFP-Rab4b was determined by light microscopy using a Nikon spinning-disk microscope equipped with a 40x water immersion objective (C3M imaging facility, Nice). The percentage of cells expressing GFP-Rab4b was obtained using the Nikon General analysis 3 software.

#### Transferrin uptake

Freshly isolated mouse hepatocytes were incubated in William’s media complemented with Alexa fluor 647-labeled transferrin (Jackson ImmunoResearch, #015-600-050) for 20 min at 37 °C. Cells were washed three times at 4 °C in William’s media without transferrin. Next, hepatocytes were centrifuged in a 96-wells plate dedicated to imaging (Greiner Bio-One, #655090) and fixed for 20 min with 4% PFA at room temperature. Images were randomly acquired in each wells using a Nikon spinning-disk microscope equipped with a 40x water immersion objective (C3M imaging facility, Nice). Quantitative information (number, area, intensity and distance distribution) was automatically extracted from images using the Nikon General analysis 3 software.

### Electron microscopy

Electron microscopy was performed at the GIS-IBiSA-labeled “Microscopie Imagerie Cytométrie Azur” MICA platform of the “Center Commun de Microscopie Appliquée” (CCMA)^66,67^. Small liver pieces harvested from Rab4b^fl/fl^ and Rab4b^HepKO^ mice were fixed overnight in 2.5% glutaraldehyde (Electron Microscopy Sciences) in 0.1 M cacodylate buffer at room temperature. Primary hepatocytes isolated from livers of Rab4b^fl/fl^ and Rab4b^HepKO^ mice were fixed overnight in 1.6% glutaraldehyde (Electron Microscopy Sciences) in phosphate buffer (pH7.4) at room temperature. Then, tissues and cells were post-fixed in potassium ferrocyanide-reduced osmium tetroxide (Electron Microscopy Sciences) for 1 h and dehydrated with increasing concentrations of acetone or ethanol respectively. Cells were incubated with a mixture of epon:ethanol (1:2 and 2:1) and finally embedded with pure epon (Electron Microscopy Sciences). Sections of 70 nm were obtained from at least three samples per conditions, using a Leica ultramicrotome and counter-stained with uranyl acetate and lead citrate. Images of at least 30 cells per conditions were acquired with a TEM JEOL1400. The numbers of early endosomes, late endosome, lysosomes and autophagic compartments-containing glycogen, easily recognizable by morphology, were counted manually for each image. The density was calculated by dividing, for each picture, the number of organelles by the manually segmented cytoplasmic area (in µm^2^), providing the number of organelles per µm^2^. Organelles were manually segmented with Fiji software (ImageJ, v.2.0.0-rc-69/1.52p), providing morphological descriptors including area and perimeter.

### RNA analysis

#### RT-qPCR

RNAs were prepared from snap-frozen tissues using trizol and RT-qPCR were performed. The qPCR reactions were performed using StepOne devices from Applied Biosystems (Thermo Fisher Scientific, Illkirch, France), using primers targeting Rab4b (Fw: AGA CCA GGG CCG TTT GG; Rv: GCC TCC AGG TAC TAA ATA ACA GAC TTG) and Stbd1 (Fw: CTG AGC CCT TCC GAT C; Rv: GCA TTG ACC CAG TCT GCT CCA A). The PCR data were normalized against mouse Rplp0 (Fw: TCC AGG CTT TGG GCA TCA; Rv: TT TAT CAG CTG CAC ATC ACT CA) gene expression and the comparative Ct (ΔΔCt) quantitation method was used^20^.

#### RNAseq

Liver tissues were lysed in trizol and sent to BGI genomics. BGI genomics extracted the RNAs, generated the libraries, performed the sequencing, the alignment and the normalization (Fragments Per Kilobase Million). The statistical analyses of RNAseq were performed with Phantasus freeware considering the differentially expressed genes as highly significant when Qvalue < 0.1, and significant when Pvalue < 0.01 (https://artyomovlab.wustl.edu/phantasus/). Clustering analyses and comparison of expression were made on Phantasus by using respectively the K-mean clustering tool and the LIMA differential expression tool. The number of K-mean clusters was determined on R-software by using the Elbow methodology. Principal Component Analysis (PCA) and Sparse Partial Least Squares Discriminant Analysis (sPLS-DA) were generated on Metaboanalyst 4.0 freeware (https://www.metaboanalyst.ca/). Colored ellipses represent a confidence of 95%. Functional explorations by gene set enrichment were performed using the free online software ShinyGo V0.76 (http://bioinformatics.sdstate.edu/go/). Values for the different Venn Diagrams were obtained by using a free online tool (https://bioinformatics.psb.ugent.be/webtools/Venn/).

Exploitation of published datasets: We analysed the level of Rab4b expression in the liver of fasted and fed Balb/C mice by using the previously published dataset GSE137385^68^. We analysed the expression of Rab4b in mice with chronic hyperglycemia (streptozotocin-induced type1 diabetes) by using the previously published dataset GSE39752^21^. Expression in human patient with NASH/MASH was performed using previously published datasets^69,70^.

### Protein analysis

WesternBlot: Snap frozen liver tissues, or primary hepatocytes, were lysed in a buffer containing 20 mM Tris, 150 mM NaCl, 10 mM EDTA, 100 mM NaF, 10 mM Pyrophosphate, 2 mM Sodium orthovanadate, complete protease inhibitor cocktail (Roche, #11836145001), and 2% Triton X-100. Protein levels in lysates were determined using Pierce BCA protein assay kit (ThermoScientific, #23225). Lysates were mixed with loading buffer and heated at 95 °C for 5 minutes before being separated by SDS-PAGE according to their molecular weight. Proteins were subsequently transferred from the gel onto an Immobilon PVDF membrane (Millipore, #IPVH00010). Immunoblotting of the membrane using the following antibodies was performed in a buffer containing 1x TBS (Euromedex, #ET220-B), 0.1% Tween-20 and 5% BSA. Primary antibodies: Hsp90 (Santa-cruz sc-13119, Mouse, 1/2'000), Erk2 (Santa-cruz, #sc1674, Mouse, 1/10’000), pAkt^Ser473^ (Cell Signaling Technology, #4060, Rabbit, 1/10’000), pAkt^Thr308^ (Cell Signaling Technology, #13038, Rabbit, 1/10’000), pGlycogen Phosphorylase^Ser15^ (Abcam, #ab227043, Rabbit, 1/10’000), tGlycogen Phosphorylase (Sigma, #HPA004119, Rabbit, 1/10’000), LC3B (Cell signaling, #3868 s, Rabbit, 1/10’000), Stbd1 (Proteintech, #11842-1-AP, Rabbit, 1/5’000), Gabarapl1 (Proteintech, #11010-1-AP, Rabbit, 1/1’000), pAMPK (Cell signaling, #2531, Rabbit, 1/1’000), p-p70S6K (Cell signaling, #9205, Rabbit, 1/1’000), SNAP-Tag (New England Biolabs, #P9310S, Rabbit, 1/1’000), and Transferrin receptor (Tfr1 / CD71 – ThermoFisher Scientific, #13-6800, Mouse, 1/2’500). Secondary antibodies: anti-Rabbit-HRP (Jackson ImmunoResearch, #715-035-152) and anti-Mouse-HRP (Jackson ImmunoResearch, #715-035-150). Detection was made using ECL (Millipore, Molsheim, France) and incremental images were acquired on an Pxi4 GeneSys Imaging system and quantifications were performed with Multi Gauge V3.0 software. Uncropped/unprocessed scans of the blots are provided in the Source Data file.

### Histology

Histological analyses were performed on the HistoC3M platform (https://www.c3m-nice.fr/plateformes/histologie/), a histology facility fully equipped by fundings from the Canceropole PACA. Mice livers were fixed in 4% paraformaldehyde overnight at 4 °C. Fixed tissues were dehydrated in increasing ethanol concentration (70%, 90%, 100%) and incubated with xylene using the automated STP120 Spin tissue Processor. Tissues were embedded in wax, and 7µm sections were collected on a microtome HM340E equipped with the “Section transfer system” (Microm Microtech). Sections were collected on glass slides. Sections were dewaxed, rehydrated, stained for Haematoxylin and Eosin and newly dehydrated using the autostainer Myreva (Microm Microtech). After mounting with a xylen-based mounting medium, images were acquired on a Nikon DS-L3 microscope equipped with 20x and 40x objectives and a digital color camera.

### Glycogen dosage

For mice liver, snap frozen liver tissues were weighted and lysed in ultrapure water using Precellys (Bertin Technologies), heated to 100 °C for 5 min and sonicated. For primary mouse hepatocytes, cells were scraped in ultrapure water, heated to 100 °C for 5 min and sonicated. Lysates were centrifuged, and Glycogen content were quantified using a Glycogen Assay kit (Sigma MAK016-1KT).

For primary human hepatocytes, cells were lysed and briefly sonicated^65^. Glycogen contents were quantified using a Glycogen Assay kit (ab65620, Abcam, Cambridge, UK).

### Glycogen phosphorylase activity assay

Livers were weighted (~50 mg) and homogenized in 500 µl TES buffer (Tris 20 mM, EDTA 1 mM, sucrose 225 mM, PMSF 0.1 mM, DTT 2.5 mM, leupeptin 1 µg/mL, aprotinin 1 µg/mL) using Precellys (Bertin Technologies). Lysates were centrifuged (16’000 g, 10 min, 4 °C) and supernatants were aliquoted. The amount of protein in the lysates was determined by BCA assay (ThermoScientific Pierce BCA kit). 500 µg of protein was incubated with an enzymatic reagent (50 mM K_2_H_2_PO_4_, 10 mM MgCl2, 5 mM EDTA, 0.5 mM NADP + , 1.5 U/mL glucose-6-phosphate dehydrogenase, 1 U/mL phosphoglucomutase and 0.1 mg/mL glycogen) for 20 min at 37 °C^71^. The reaction was stopped on ice, and the remaining NADP+ was degraded by heating the samples at 60 °C for 30 min. The final product of these successive enzymatic reactions is the NADPH, and the activity of the endogenous glycogen phosphorylase is rate limiting for this production. Therefore, NADPH content was quantified using a NADP/NADPH Assay kit (Sigma, #MAK479).

### Flow cytometry

Liver cells were isolated following the procedure described above (primary mouse hepatocyte isolation) until the step of filtration through 100 µm filters. After this step, cells were centrifuged at 50 g for 5 min, and the supernatant, containing the non-parenchymal cells, was collected. Non-parenchymal cells were washed and incubated at 4 °C for 30 min with either CD45-APC-Cy7 (clone 30-F11, 1/200, Biolegend), CD11b-BV510 (clone M1/70, 1/200, Biolegend), Clec2-PE (clone 17D9, 1/200, Biolegend), CD206-PerCP-Cy5.5 (clone C068C2, 1/200, Biolegend), CD301-FITC (clone ER-MP23, 1/200, BioRad), Timd4-PE-Cy7 (clone RMT4-54, 1/200, Biolegend) and MHCll-APC (clone M5/114.15.2, 1/200, Biolegend) antibodies, or CD79β-PE (clone HM79-12, 1/200, Biolegend), TCRβ-PB (clone H57-597, 1/200, Biolegend), NK1.1-FITC (clone S17016D, 1/200, Biolegend), Ly6C-APC (clone HK1.4, 1/200, Biolegend), and Ly6G-PerCP-Cy5.5 (clone 1A8, 1/200, Biolegend) antibodies. Flow cytometry analysis was then performed on a BD Canto II (C3M flow cytometry facility).

### Statistical analysis

Data are presented as the mean ± SEM. GraphPad PRISM5 software was used. For statistical significance between two groups, we first evaluated whether the distribution was symmetrical or asymmetrical using the Shapiro-Wilk normality test. For normal distribution, we applied Student T-test without the Welch correction for homogenous variance, or with Welch correction when the variance was found heterogeneous using the F-test. For asymmetrical distribution, we applied Mann & Whitney test. For statistical significance between more than two groups, we performed one-way ANOVA. For statistical significance between two independent variables, we performed two-way ANOVA. All statistical details of experiments can be found in the figure legends.

### Reporting summary

Further information on research design is available in the Nature Portfolio Reporting Summary linked to this article.

## Supplementary information

Supplementary Information

Reporting Summary

Transparent Peer Review file

## Source data

Source Data

## Data Availability

The RNA-seq data generated in this study have been deposited in the NCBI’s Gene Expression Omnibus database under accession code GSE333473. In addition, we used the following published datasets: GSE137385, GSE39752, GSE110404 and GSE130970. All data supporting the findings described in this manuscript are available in the article, in the Supplementary Information/Source Data file, and from the corresponding authors upon request. Source data are provided with this paper.
