# Supplementary material for: Rab4b controls hepatic glucose production during fasting through glycophagy
Source: Nat Commun. 2026 Aug 10;17:9587. doi: 10.1038/s41467-026-76218-8 (PMC13550640; doi:10.1038/s41467-026-76218-8)
Supplement: Supplementary file 1 [file 41467_2026_76218_MOESM1_ESM.pdf]

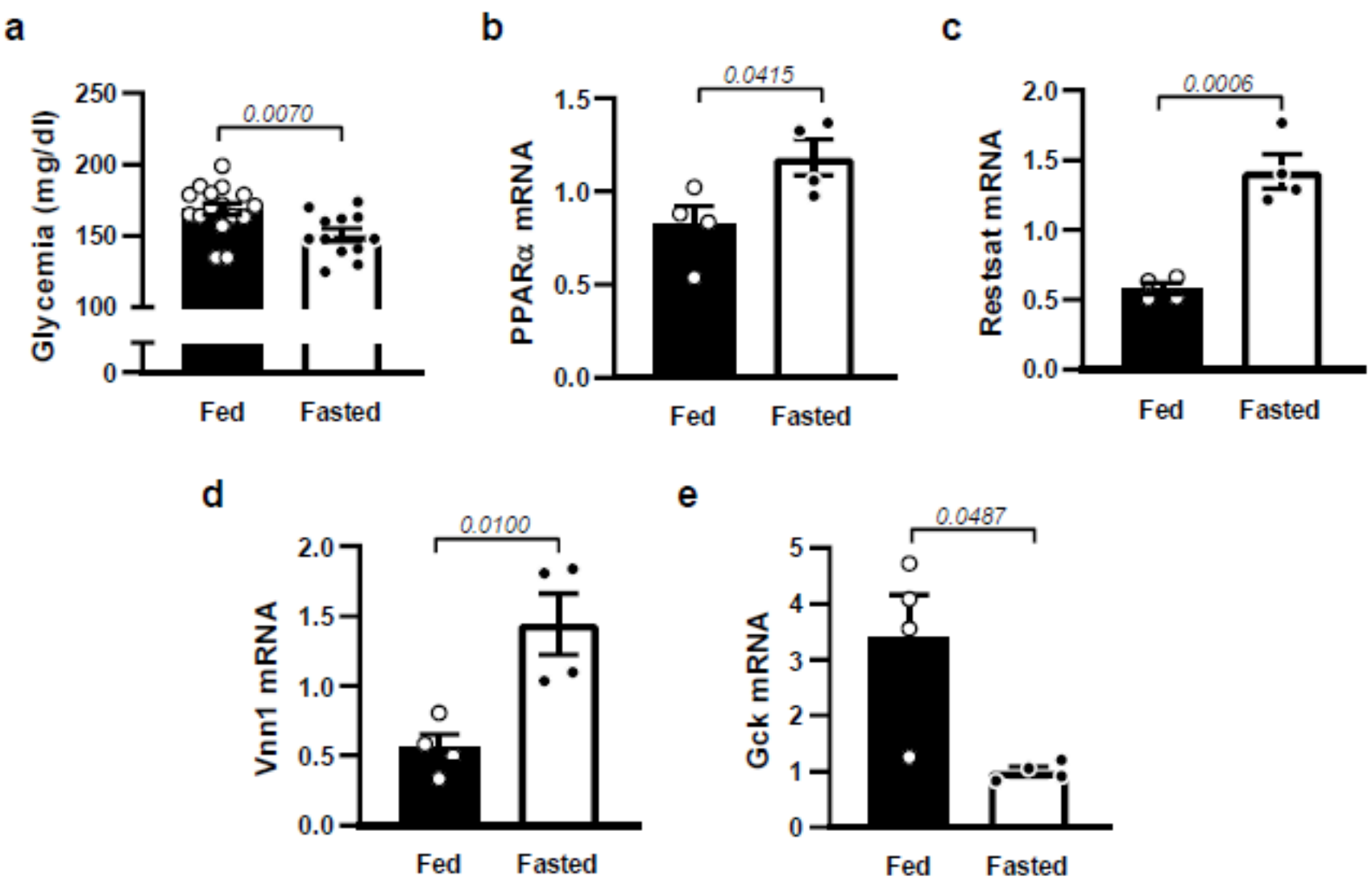

Supplementary Figure 1. Metabolic and transcriptomic data of the fasting/feeding mice model. **(a)** Glycemia in fed (n=16) or fasted (n=12) C57Bl6 mice. **(b)** mRNA expression of PPARα in the liver from fed or fasted C57Bl6 mice (n=4/condition). **(c)** mRNA expression of the PPARα target gene Retsat in the liver from fed or fasted C57Bl6 mice (n=4/condition). **(d)** mRNA expression of the PPARα target gene Vnn1 in the liver from fed or fasted C57Bl6 mice (n=4/condition). **(e)** mRNA expression of glucokinase (Gck) in the liver from fed or fasted C57Bl6 mice (n=4/condition). Error bars indicate means ± s.e.m. Unpaired two-sided t-test was used for panels (a-d), and unpaired two-sided t-test with Welch's correction for panel (e). Source data are provided as a Source Data file.

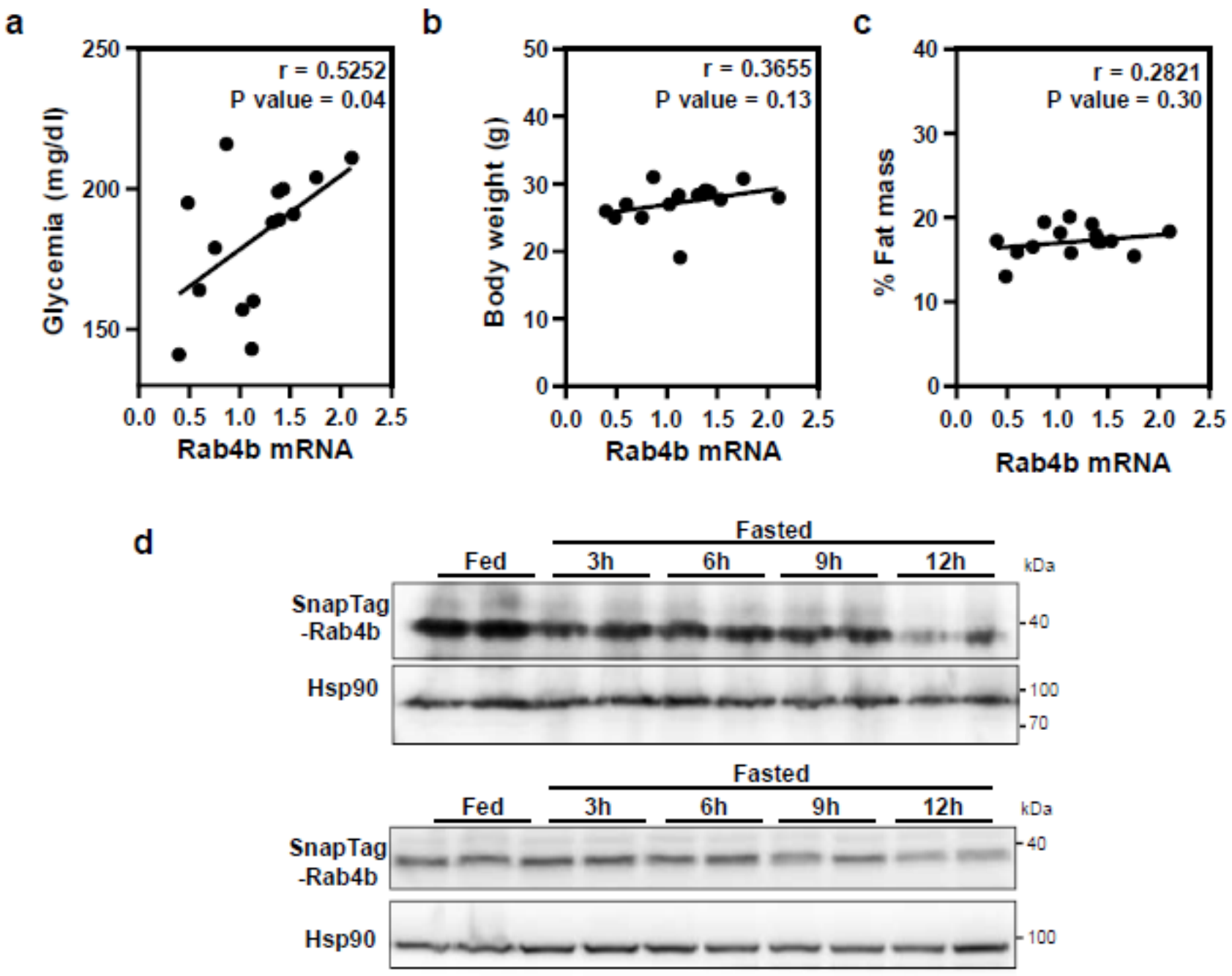

e

	Fold change	PValue	FDR
NAFL vs Normal_weight	2,747067817	2,92E-08	6,67E-07
NASH vs Normal_weight	2,652113953	5,06709E-08	8,10056E-07

Supplementary Figure 2. Rab4b mRNA and protein levels in liver. (a) Correlation between liver Rab4b mRNA expression and glycemia in fasted and fed C57/Bl6 mice (n=15). **(b)** Correlation between liver Rab4b mRNA expression and body weight in fasted and fed C57/Bl6 mice (n=15). **(c)** Correlation between liver Rab4b mRNA expression and % of fat mass in fasted and fed C57Bl6 mice (n=15). **(d)** Western blot of snaptag-Rab4b on liver lysates from the Snaptag-Rab4b mice in fed and fasted conditions (n=4/conditions). Hsp90 is used as loading control. **(e)** Rab4b mRNA expression fold change between livers from healthy versus NAFLD or NASH patients. Extracted from a published dataset[70]. Error bars indicate means ± s.e.m. Pearson correlation was used for panels (a-c). Source data are provided as a Source Data file.

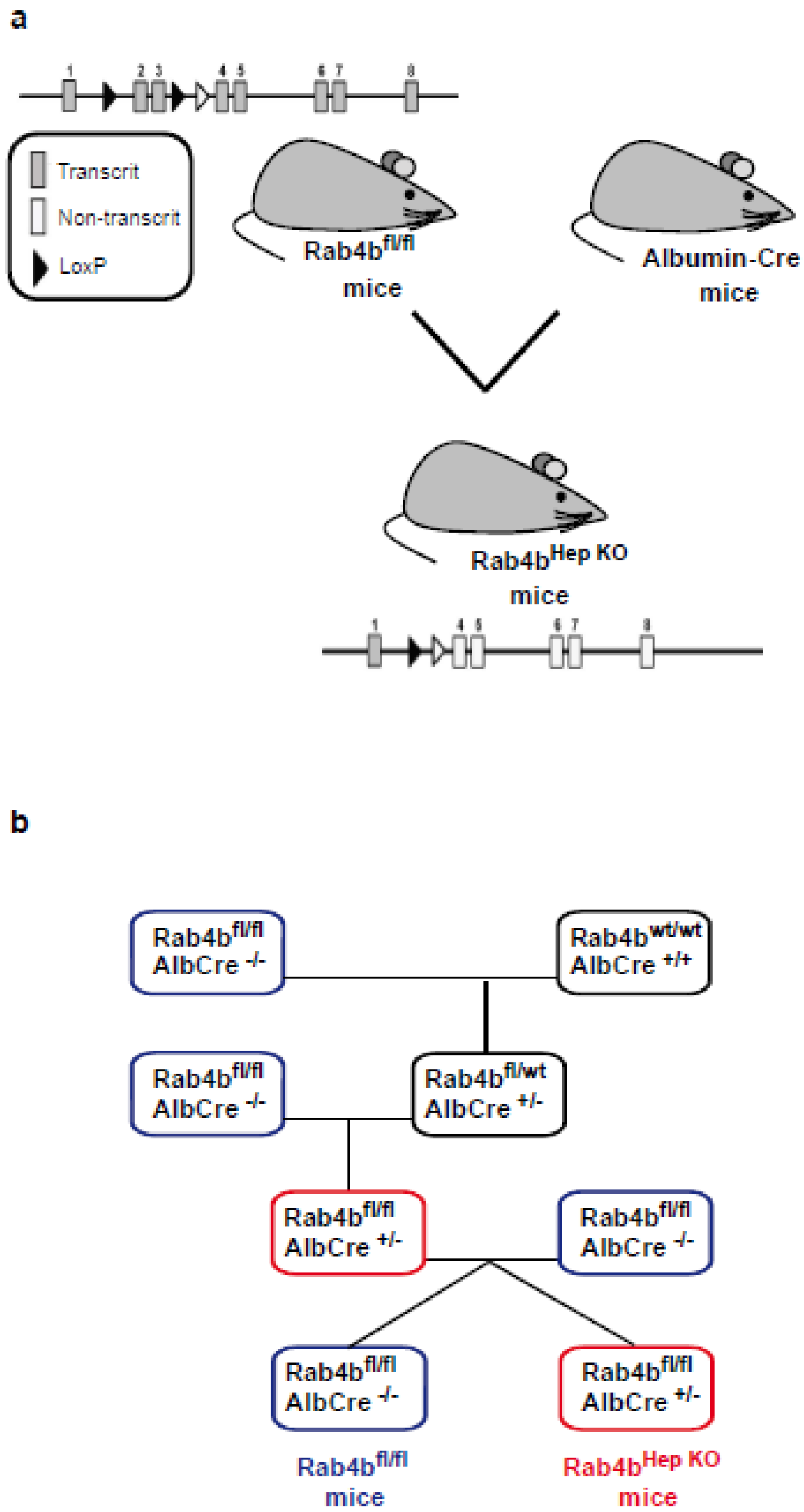

Supplementary Figure 3. Breeding strategy to produce Rab4b^{HepKO} mice. (a) Schematic illustration of the strategy to obtain a hepatocyte specific knock-out of Rab4b. **(b)** Breeding strategy to obtain Rab4b$^{fl/fl}$ and Rab4b^{HepKO} littermate mice.

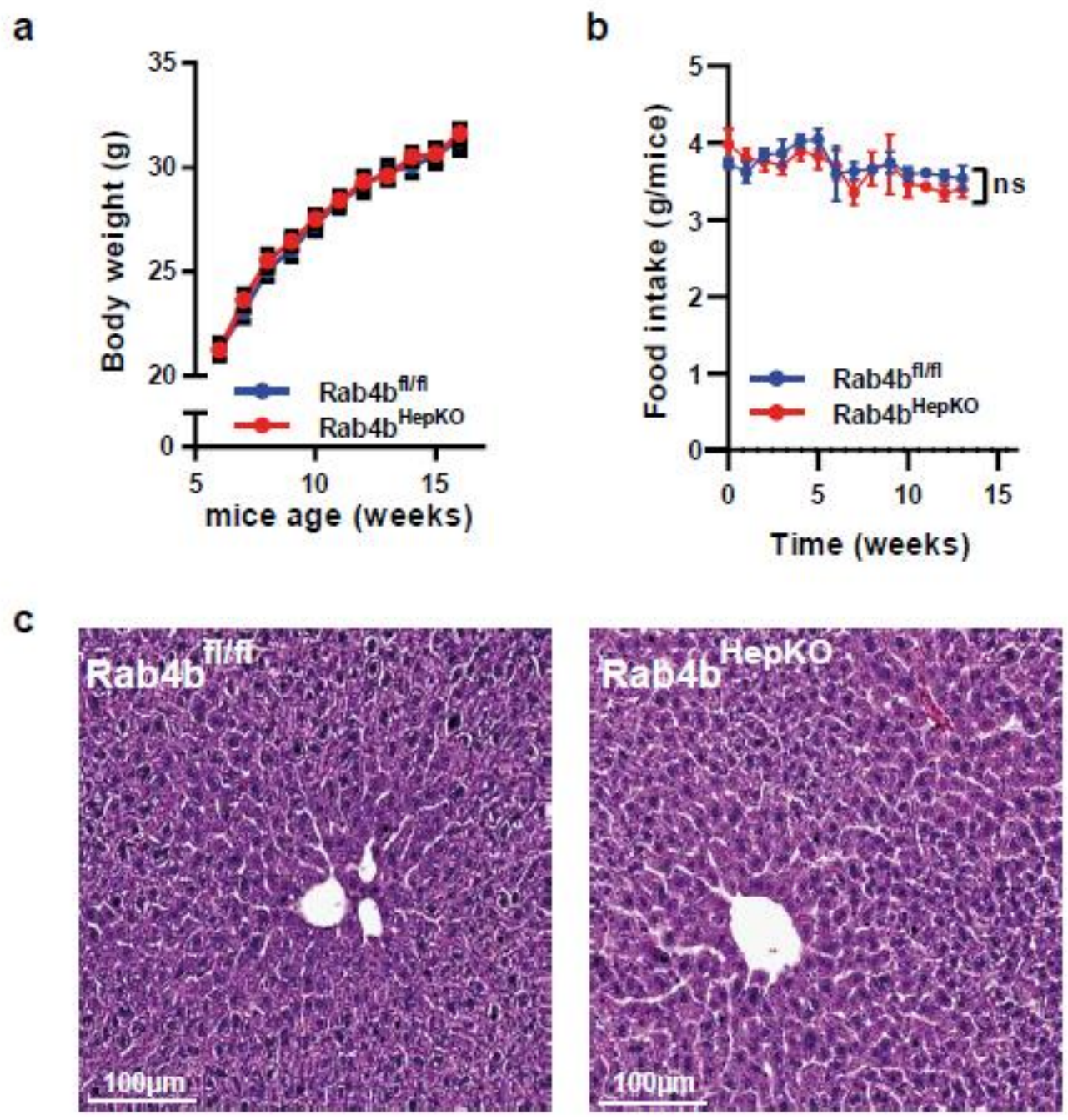

Supplementary Figure 4. Body weight, food intake and liver histology of Rab4b^{HepKO} mice. (a) Evolution of body weight in Rab4b$^{fl/fl}$ and Rab4b^{HepKO} mice under chow diet (n=12/genotype). **(b)** Evolution of food intake in Rab4b$^{fl/fl}$ and Rab4b^{HepKO} mice (n=12/genotype). **(c)** Representative images of liver sections from Rab4b$^{fl/fl}$ and Rab4b^{HepKO} mice stained with hematoxylin & eosin (n=12/genotype). Error bars indicate means ± s.e.m. Two-way ANOVA was used for panel (b). Source data are provided as a Source Data file.

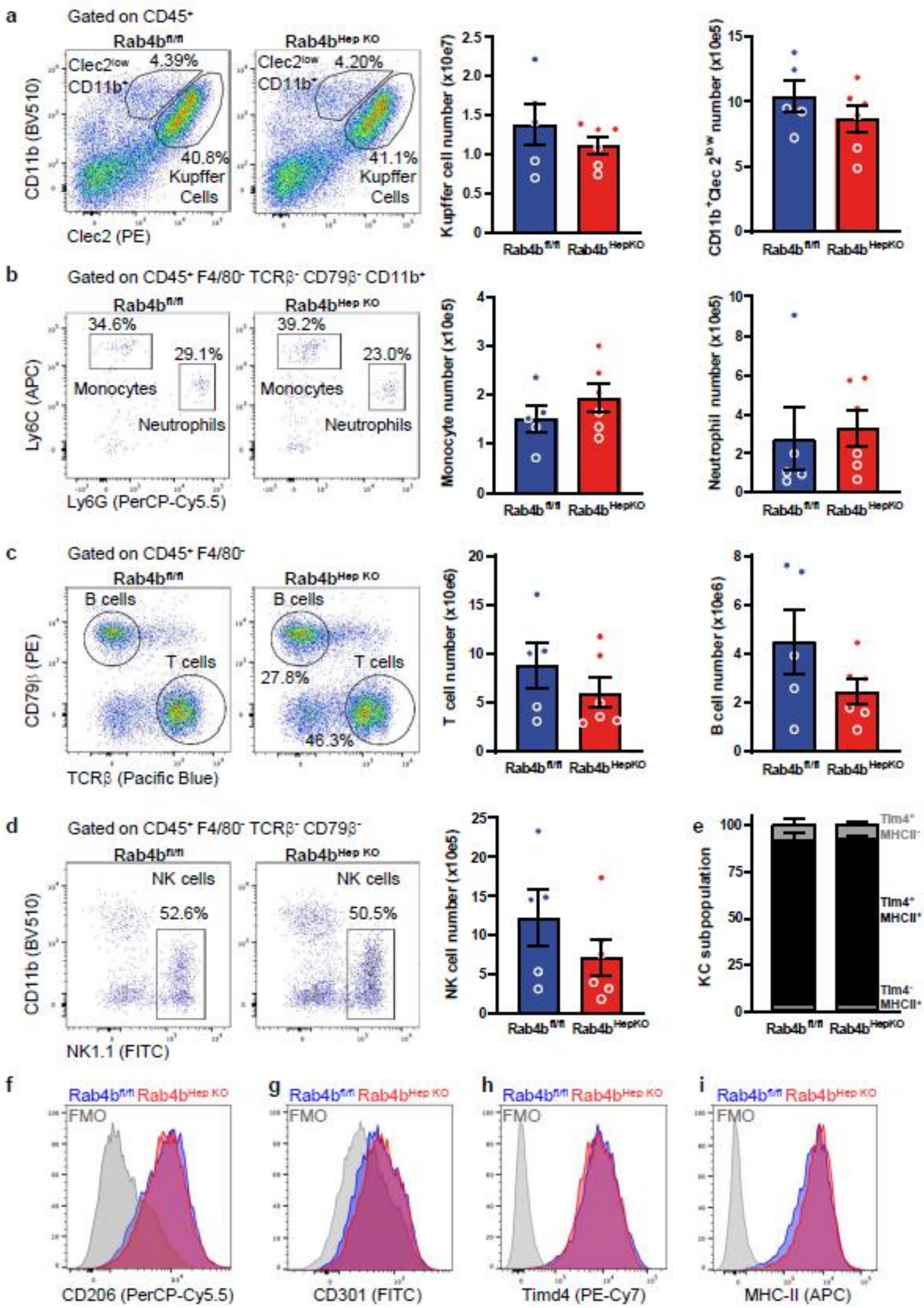

Supplementary Figure 5. Non-parenchymal cell populations in the liver of Rab4b^{HepKO} mice. (a) Representative dot plot of flow cytometry analyses of CD45^{+} liver cells from fasted Rab4b$^{fl/fl}$ and Rab4b^{HepKO} mice gated with CD11b and Clecl2 markers (left panels). The surrounded area represents respectively Clecl2^{low}/CD11b^{+} and Kuppfer cell populations. Quantification of these populations is

represented as bar graphs (right panels). **(b)** Representative dot plot of flow cytometry analyses on CD45$^+$ F4/80$^-$ TCRβ$^-$ CD79β$^-$ CD11b$^+$ liver cells from fasted Rab4b$^{fl/fl}$ and Rab4b^{HepKO} mice gated on Ly6C and Ly6G markers (left panels). The surrounding area represents respectively monocytes and neutrophils. Quantification of these populations is represented as bar graphs (right panels). **(c)** Representative dot plot of flow cytometry analyses on CD45$^+$ F4/80$^-$ liver cells from fasted Rab4b$^{fl/fl}$ and Rab4b^{HepKO} mice gated on CD79β and TCRβ markers (left panels). The surrounding area represents respectively B and T cells. Quantification of these populations is represented as bar graphs (right panels). **(d)** Representative dot plot of flow cytometry analyses on CD45$^+$ F4/80$^-$ TCRβ$^-$ CD79β$^-$ liver cells from fasted Rab4b$^{fl/fl}$ and Rab4b^{HepKO} mice gated on CD11b and NK1.1 markers (left panels). The surrounding area represents NK cells. Quantification of this population is represented as bar graphs (right panels). **(e)** Quantification of the different Kuppfer cell subpopulations. **(f-i)** Histograms of the surface expression of the indicated markers. Unstained is represented in grey color. For all panels n=5 Rab4b$^{fl/fl}$ mice and 6 Rab4b^{HepKO} mice. Error bars indicate means ± s.e.m. Source data are provided as a Source Data file.

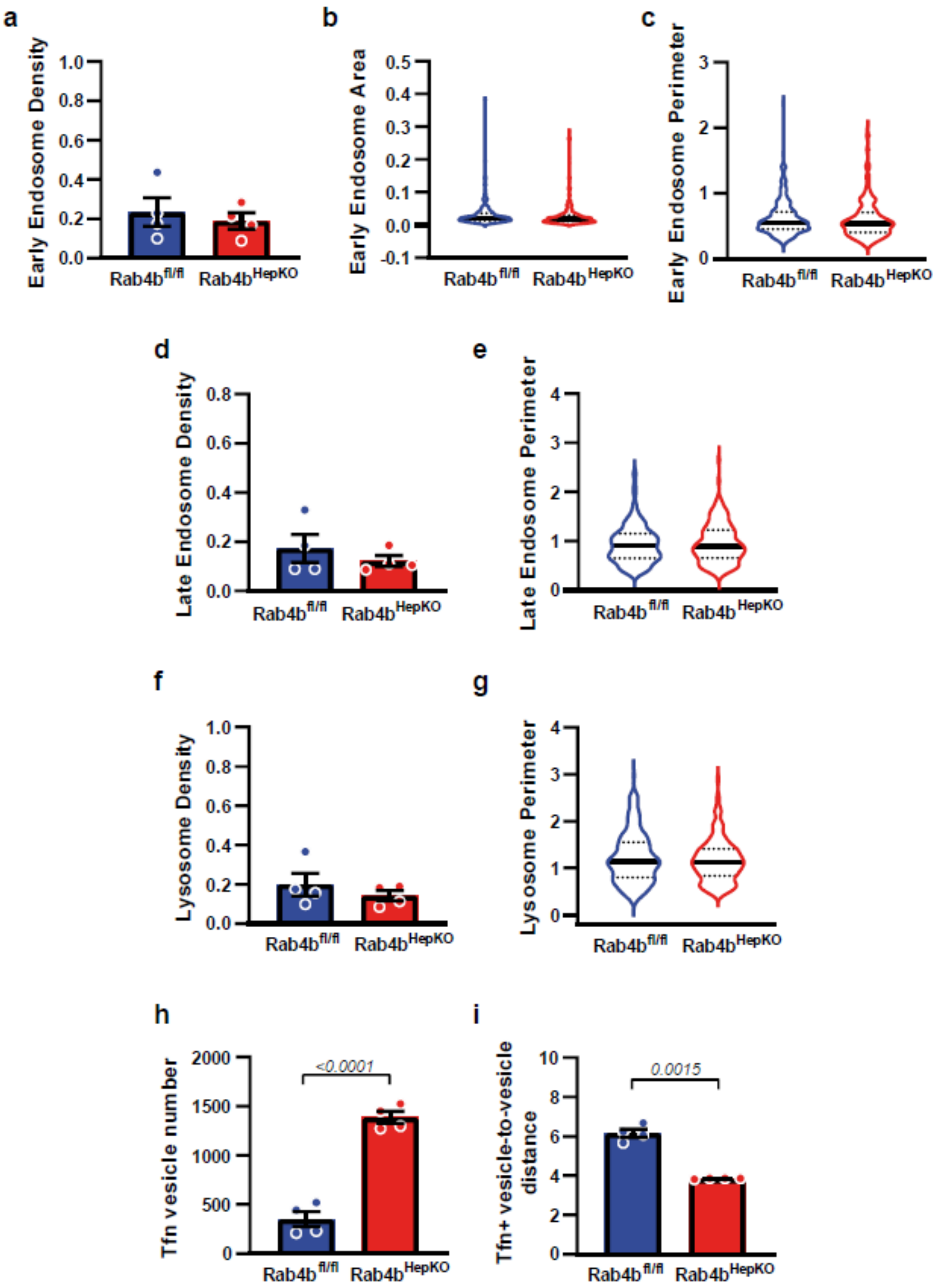

Supplementary Figure 6. Endocytic compartments and transferrin recycling in the liver of Rab4b^{HepKO} mice. (a) Quantification of early endosome density (number/ µm^2) in electron microscopy sections of livers from random-fed Rab4b$^{fl/fl}$ and Rab4b^{HepKO} mice (n=4/genotype). **(b,c)** Quantification of early endosome area (b - µm^2) and perimeter (c - µm) in electron microscopy sections of livers from random-fed Rab4b$^{fl/fl}$ and Rab4b^{HepKO} mice (n=258 for Rab4b$^{fl/fl}$ and 216 for Rab4b^{HepKO} mice). **(d)** Quantification of late endosome density (number/ µm^2) in electron microscopy sections of livers from random-fed Rab4b$^{fl/fl}$ and Rab4b^{HepKO} mice (n=4/genotype). **(e)** Quantification of late endosome perimeter (µm) in electron microscopy sections of livers from random-fed Rab4b$^{fl/fl}$ and Rab4b^{HepKO} mice (n=165 for Rab4b$^{fl/fl}$ and 138 for Rab4b^{HepKO} mice). **(f)** Quantification of lysosome density (number/ µm^2) in electron microscopy sections of livers from random-fed Rab4b$^{fl/fl}$ and Rab4b^{HepKO} mice (n=4/genotype). **(g)** Quantification of lysosome perimeter (µm) in electron microscopy sections of livers from random-fed Rab4b$^{fl/fl}$ and Rab4b^{HepKO} mice (n=210 for Rab4b$^{fl/fl}$ and 170 for Rab4b^{HepKO} mice). **(h,i)** Quantification of steady-state transferrin positive vesicle number (h) and mean vesicle-to-vesicle distance (i) in random-fed primary hepatocytes freshly isolated from Rab4b$^{fl/fl}$ and Rab4b^{HepKO} mice (n=4/genotype). Error bars indicate means ± s.e.m. Unpaired two-sided t-test was used for panel (h), and unpaired two-sided t-test with Welch's correction for panel (i). Source data are provided as a Source Data file.

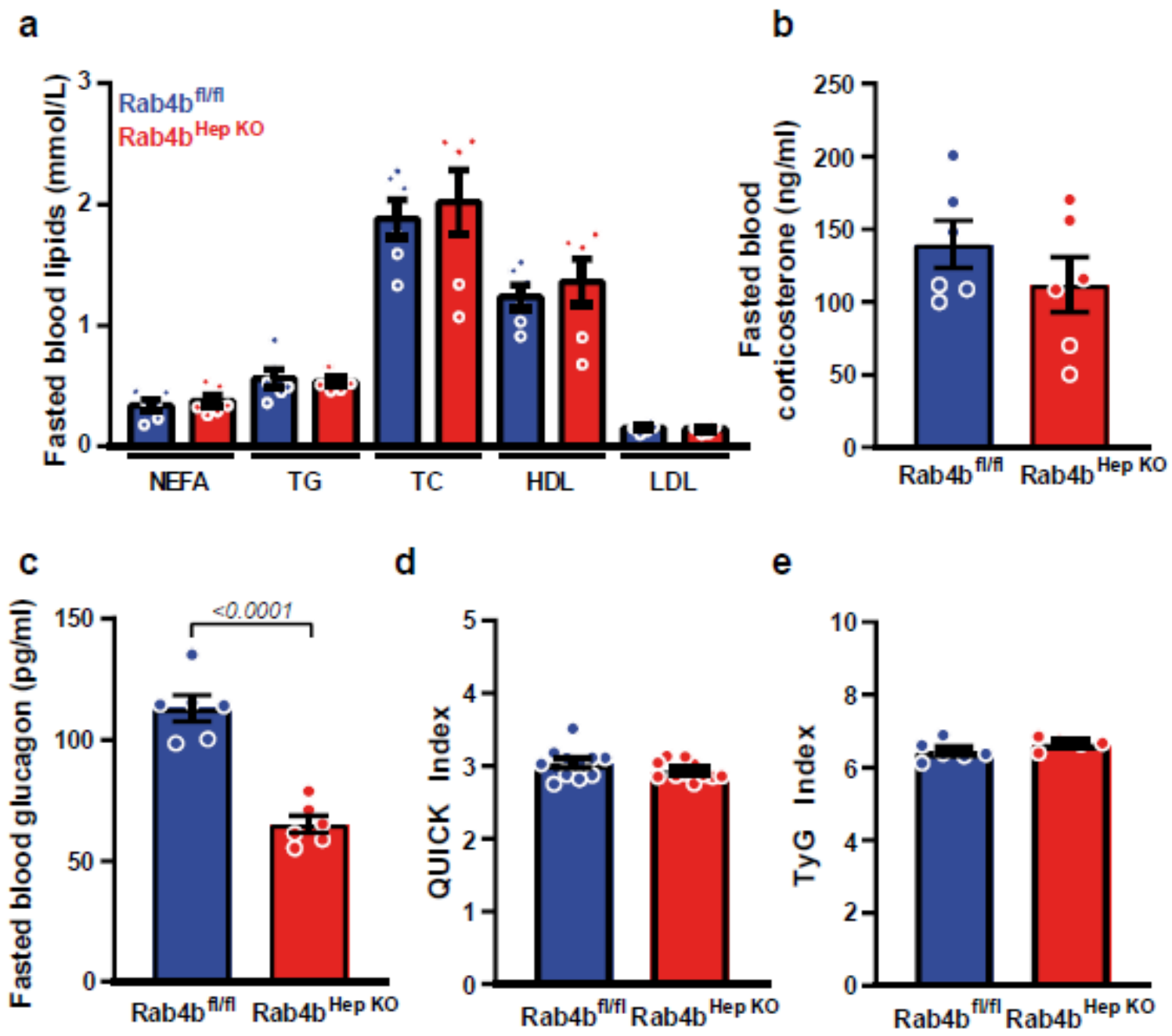

Supplementary Figure 7. Circulating lipids and insulin-resistance test in Rab4b^{HepKO} mice. (a) Circulating levels of non-esterified fatty acids (NEFA), triglycerides (TG), total cholesterol (TC), high-density lipoprotein (HDL) and low-density lipoprotein (LDL) in 6 h-fasted Rab4b$^{fl/fl}$ and Rab4b^{HepKO} mice (n=6/genotype). **(b)** Circulating levels of corticosterone in 6h-fasted Rab4b$^{fl/fl}$ and Rab4b^{HepKO} mice (n=6/genotype). **(c)** Circulating levels of glucagon in 6 h-fasted Rab4b$^{fl/fl}$ and Rab4b^{HepKO} mice (n=6/genotype). **(d)** Quantitative insulin sensitivity check index (QUICK index) in Rab4b$^{fl/fl}$ and Rab4b^{HepKO} mice (n=11/genotype). **(e)** Triglycerides-Glucose index (TyG index) in Rab4b$^{fl/fl}$ and Rab4b^{HepKO} mice (n=6 Rab4b$^{fl/fl}$ and 5 Rab4b^{HepKO} mice). Error bars indicate means ± s.e.m. Unpaired two-sided t-test was used for panel (c). Source data are provided as a Source Data file.

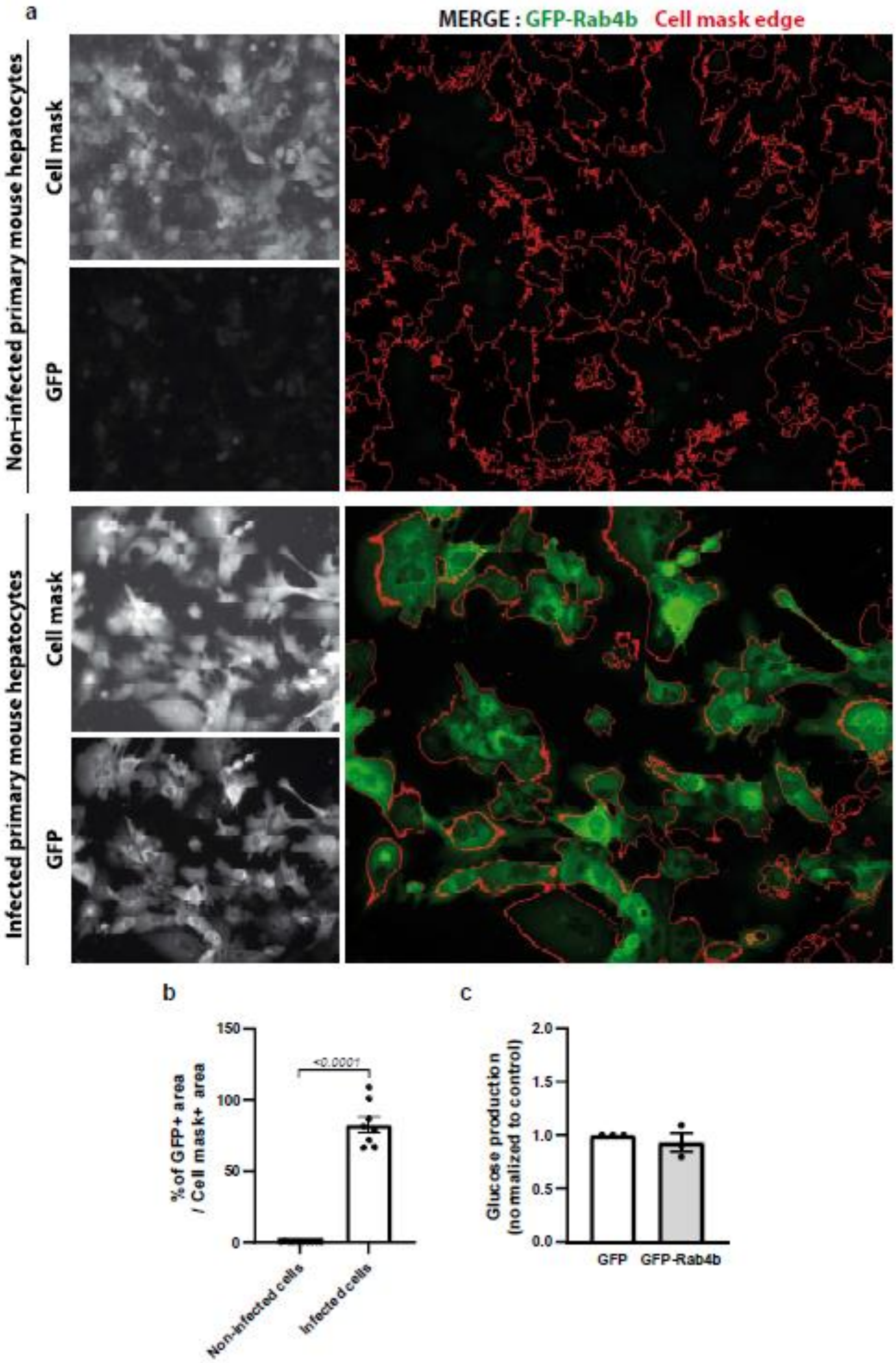

Supplementary Figure 8. Glucose production in mouse primary hepatocytes overexpressing Rab4b. (a) Images from primary mouse hepatocytes infected with GFP-Rab4b (bottom panels) compared to untreated cells (top panels). (n=8/conditions). **(b)** Quantification of the percentage of hepatocytes overexpressing GFP-Rab4b in the conditions described in (a). (n=8/conditions). **(c)** Glucose production in primary mouse hepatocytes infected with lentivirus expressing GFP or GFP-Rab4b, and treated for 4h with fasting culture media (n=3). Error bars indicate means ± s.e.m. Unpaired two-sided Mann-Whitney test was used for panel (b). Source data are provided as a Source Data file.

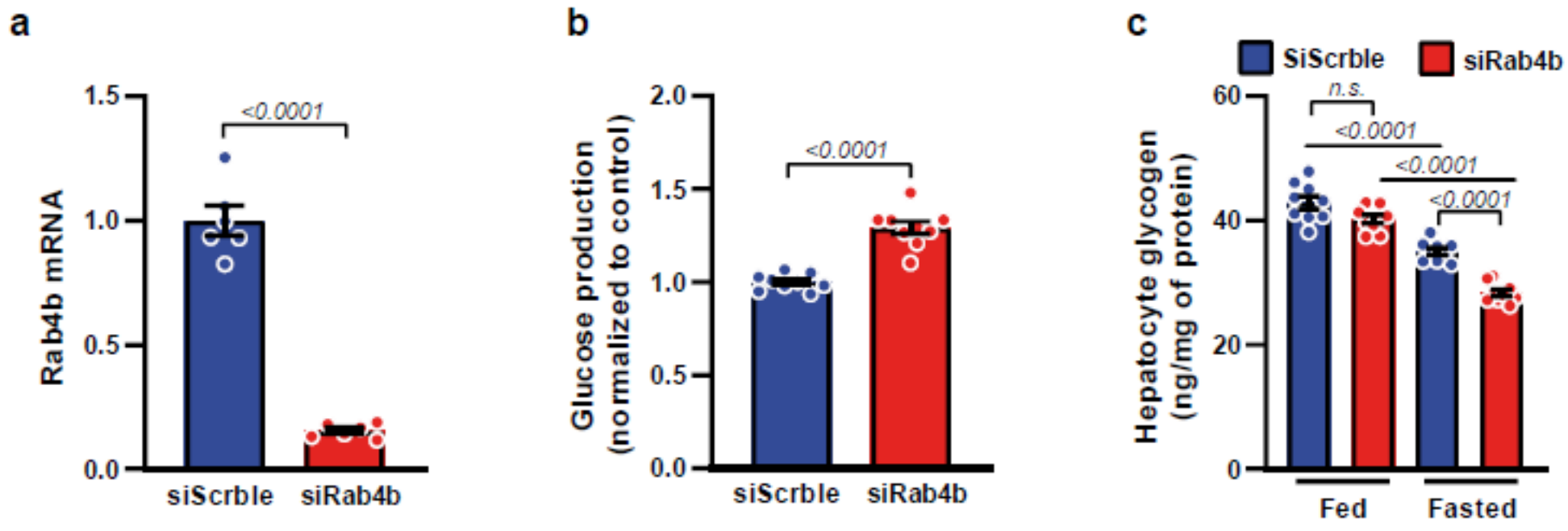

Supplementary Figure 9. Effect of Rab4b silencing in human primary hepatocytes. (a) mRNA level of Rab4b in human primary hepatocytes treated with RNAi directed against a scramble sequence (control) or Rab4b (n=6/conditions). **(b)** Glucose production in human primary hepatocytes control (siScramble) or silenced for Rab4b (siRab4b) and treated for 12 h with fasting culture media (n=9/conditions). **(c)** Glycogen level in human primary hepatocytes control (siScramble) or silenced for Rab4b (siRab4b) and treated for 12 h with feeding or fasting culture media (n=9/conditions). Error bars indicate means ± s.e.m. Unpaired two-sided t-test with Welch's correction was used for panels (a,b), and two-way ANOVA with Sidak multiple-comparisons for panel (c). Source data are provided as a Source Data file.

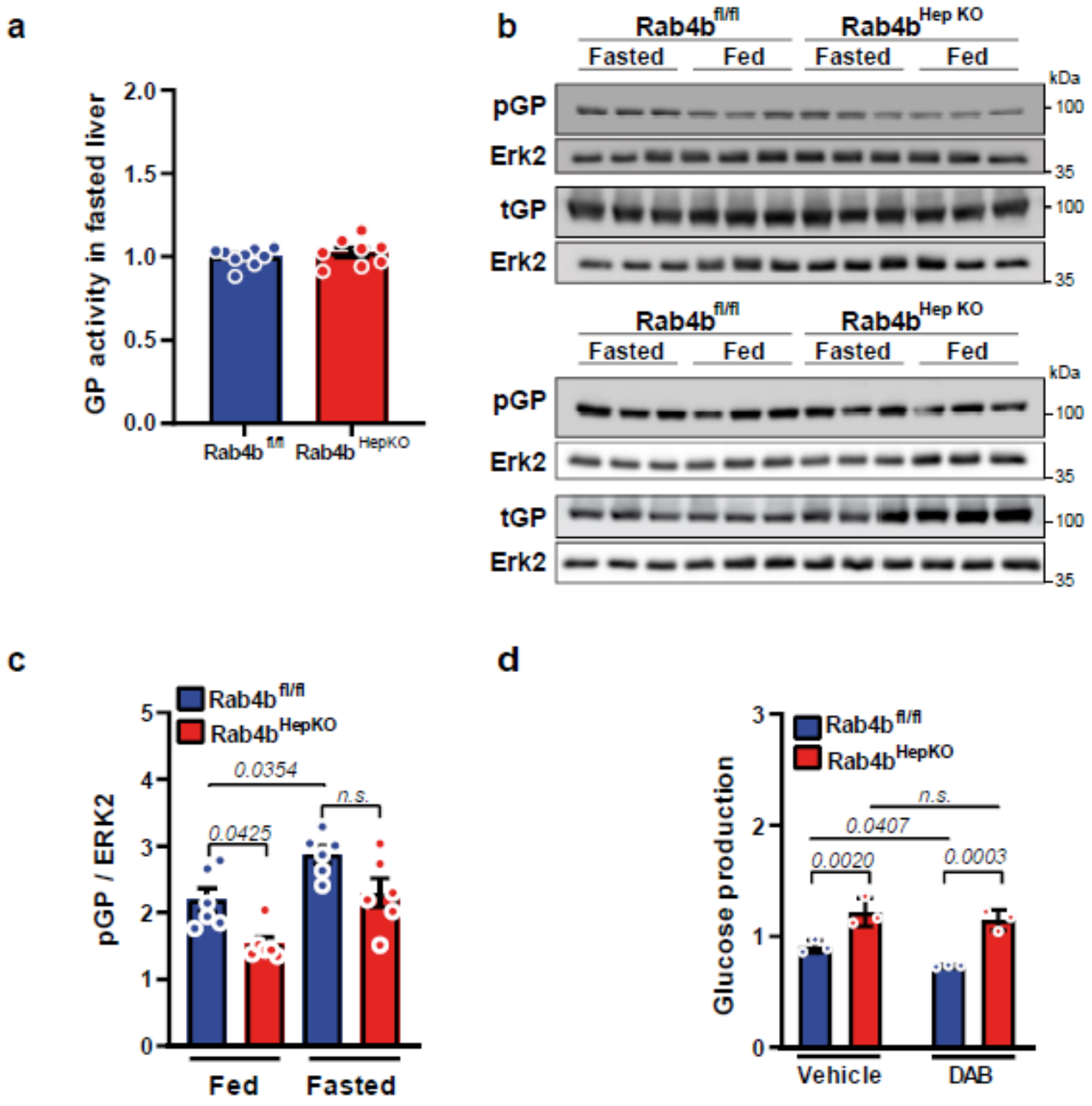

Supplementary Figure 10. Glycogenolysis in hepatocytes from Rab4b$^{fl/fl}$ Rab4b^{HepKO} mice. (a) *In vitro* glycogen phosphorylase activity assay using liver lysates from fasted Rab4b$^{fl/fl}$ and Rab4b^{HepKO} mice (n=9 Rab4b$^{fl/fl}$ and 8 Rab4b^{HepKO} mice). **(b)** Representative Western Blot of total and phosphorylated glycogen phosphorylase (tGP and pGP) in livers from fasted or fed Rab4b$^{fl/fl}$ and Rab4b^{HepKO} mice. Erk2 is used as loading control (n=6/conditions). **(c)** Quantification of phosphorylated glycogen phosphorylase level from immunoblots as presented in (b). (n=6/conditions). **(d)** Glucose production in Rab4b$^{fl/fl}$ and Rab4b^{HepKO} primary mouse hepatocytes treated for 4h with fasting culture media complemented with or without 1,4-dideoxy-1,4-imino-d-arabinitol (DAB), a glycogen phosphorylase inhibitor (n=3/conditions). Error bars indicate means ± s.e.m. Two-way ANOVA with Tukey multiple-comparisons was used for panel (c), and two-way ANOVA with Benjamini-Hochberg FDR method for panel (d). Source data are provided as a Source Data file.

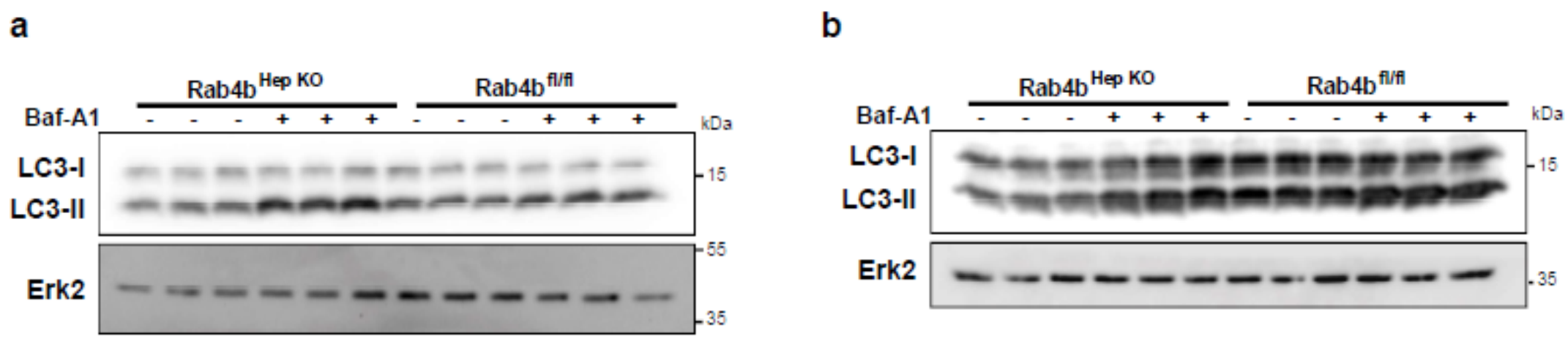

Supplementary Figure 11. Autophagy induction in Rab4b$^{fl/fl}$ and Rab4b^{HepKO} hepatocytes. (a and **b)** Western Blot of LC3 in hepatocytes treated with fasting media in presence or absence of bafilomycin-A1 used for the quantification presented in Figure 6c (n=9/ conditions). Source data are provided as a Source Data file.

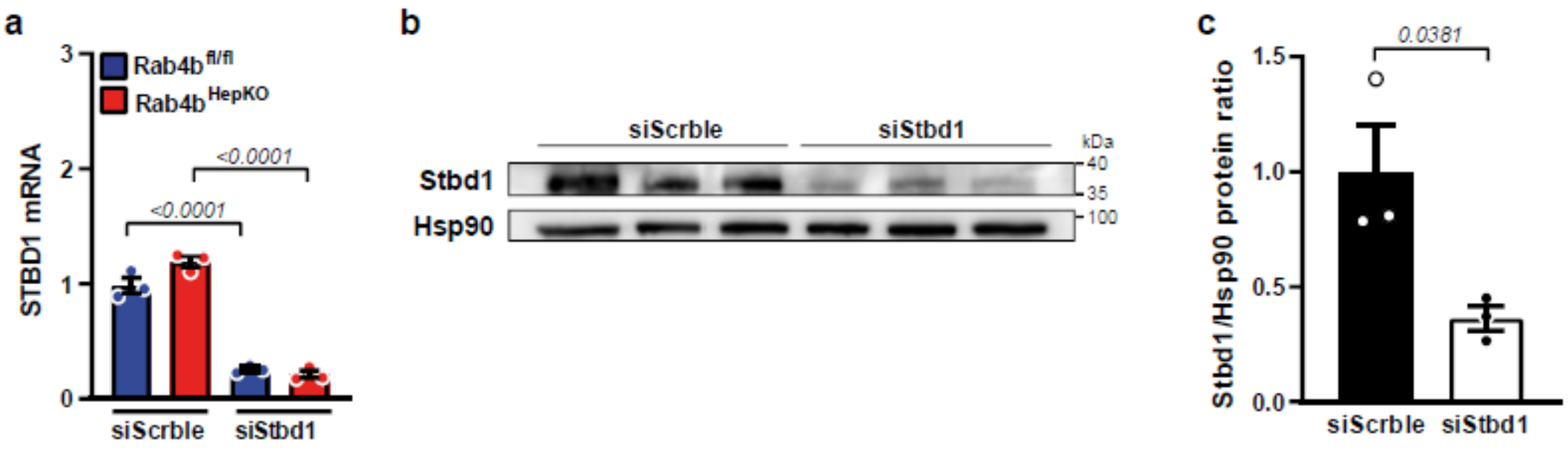

Supplementary Figure 12. Validation of Stbd1 silencing. (a) mRNA level of Stbd1 in Rab4b$^{fl/fl}$ and Rab4b^{HepKO} primary mouse hepatocytes treated with RNAi directed against a scramble sequence (control) or Stbd1 (n=3/conditions). **(b)** Representative Western Blot of Stbd1 in livers from control mice treated *in vivo* with RNAi against a Scramble sequence or Stbd1 (n=3/conditions). Hsp90 is used as loading control. **(c)** Quantification of Stbd1 level from the immunoblot presented in (b). (n=3/conditions). Error bars indicate means ± s.e.m. Two-way ANOVA with Sidak multiple-comparisons was used for panel (a), and unpaired two-sided t-test for panel (c). Source data are provided as a Source Data file.
