# Supplementary material for: Rab4b controls hepatic glucose production during fasting through glycophagy
Source: Nat Commun. 2026 Aug 10;17:9587. doi: 10.1038/s41467-026-76218-8 (PMC13550640; doi:10.1038/s41467-026-76218-8)
Supplement: Supplementary file 3 [file 41467_2026_76218_MOESM3_ESM.pdf]

nature portfolio

Peer Review File

Rab4b controls hepatic glucose production during fasting through glycophagy

Corresponding Author: Dr Jerome Gilleron

This file contains all reviewer reports in order by version, followed by all author rebuttals in order by version.

Version 0:

Reviewer comments:

Reviewer #1

(Remarks to the Author)
Dussot et al showed that hepatocyte-specific Rab4b knockout, which disrupts endocytic recycling, elicits an increased capacity to produce glucose by enhanced glycophagy. The manuscript reveals a unique role of Rab4b in glycophagy regulation, however, some of the key aspects are not sufficiently documented. Most importantly, there is scarcity of mechanistic insights into the connection among Rab4b, autophagy flux and glycophagy.
1. In Fig 1c-f, the Rab4b mRNA levels are positively correlated with blood glucose concentration. However, the manuscript showed loss of hepatic rab4b caused fasting hyperglycemia in mice. How to explain these contradicted results?
2. In Fig 1d-f, the author showed the mRNA levels of Rab4b are upregulated in STZ, WD or STAM mice models. The common feature of these mice models are insulin resistance or insulin deficiency-associated hyperglycemia. Are they also related to dysregulated glycophagy? The same question to the Fig 1g.
3. Rab4b controls several endocytic recycling pathways. The manuscript should reveal which protein's endocytic recycling mediates hyperglycemia in Rab4b hepatic knockout mice after a short-time fasting.
4. In Fig 3g the author only showed the p value of these transcriptional factors. We could not tell they were upregulated or downregulated. It is better to show the fold of alternation also.
5. The fasting blood glucose level is determined not only by insulin, but also glucagon and glucocorticoids. The author should measure these hormone levels (Fig 4c,d) in WT and Rab4b knockout mice
6. How can Rab4b deficiency increase autophagy flux (Fig 6c)? What is the molecular mechanism? For example, does rab4b co-localize to autolysosome or autophagosome? Does rab4b affect AMPK or mTORC1 pathway?
7. Although the author discuss the potential clinical relevance of targeting Rab4b to glycogen storage disease (GSDs), it is better to prove it in GSD mouse model.
8. There is no any Rab4b-overexpression or Rab4b rescure data throughout the manuscript. Does rab4b overexpression cause the reverse effects as compared to gene knockout?

Reviewer #2

(Remarks to the Author)
Reviewer Comments:
In this manuscript, the authors describe a novel role for Rab4b in glycophagy regulation in the liver. They demonstrate that loss of hepatic Rab4b leads to increased fasting plasma glucose levels within 0–9 hours of fasting. The data suggest that Rab4b acts as an inhibitor of glycophagy. Overall, the paper is very well written, and the data are clearly presented. However, I have a few points that should be addressed to further strengthen the manuscript.

Major points:
In Figure 1, the authors show that hepatic Rab4b mRNA expression is decreased after 6 hours of fasting. However, they do not provide any data on Rab4b protein levels. Since protein levels may not always correlate with mRNA expression, I recommend that the authors confirm the downregulation at the protein level. Furthermore, in the discussion, the authors propose that Rab4b is involved in an early fasting-induced autophagy wave. Do they observe similar temporal dynamics at the protein level? It would be informative to examine Rab4b protein expression at multiple time points during fasting (e.g., 3, 6, 9, and 12 hours) to better characterize its regulation.
In case total Rab4b levels are not changed, is its intracellular localization changed?

This study may have clinical implications for glycogen storage diseases, such as GSDI. Could the authors comment on whether Rab4b loss might be beneficial in the context of G6Pase deficiency? Would a dual knockdown model (e.g., siRNA-mediated suppression of both Rab4b and G6Pase) help to test this hypothesis?

All experiments appear to have been conducted in mice and primary mouse hepatocytes. To improve the translational relevance of their findings, the authors should consider replicating key results in a human-relevant model, such as human primary hepatocytes.

Although the authors show that Rab4b inhibits glycophagy, the underlying mechanism remains unclear. Can the authors provide further speculation or working hypotheses on how Rab4b may regulate glycophagy at the molecular level? Identifying potential receptors that might be affected by the loss of Rab4b. Is it initiation or autophagosome/lysosome fusion? This is relevant information to distinguish specific autophagic pathways, such as glycophagy in the early wave.

Reviewer #3

(Remarks to the Author)
This study examines how disruptions of endocytic membrane traffic in the liver impact cell and systemic metabolism. This work builds on prior studies, in part from this group, that showed an essential role for the Rab5 in maintenance of endosomes, control of many aspects of systemic metabolism, and regulation of gene expression. This current manuscript aims to understand the role of Rab4b, known to regulate distinct endosomal membrane traffic processes compared to Rab5, in liver metabolism and systemic glucose homeostasis. Fasting triggered a reduction in Rab4b mRNA, and conditions with elevated blood glucose led to an increase in Rab4b in several rodent models. The role of Rab4b was first functionally examined in a liver-specific Rab4b knockout animal model. Rab4b liver-KO animals had modest perturbations in endosome appearance, with a noted increase in the uptake of transferrin, a widely-used model of internalization and endosomal traffic. RNAseq analysis was performed in Rab4b liver-KO and wild-type animals, in both fed and fasted states. While gene expression was similar between genotypes in the fed state, the fasted states exhibited many differently expressed genes between genotypes. Various analyzes revealed specific clusters of similarly regulated genes.

In contrast to wild-type animals that exhibited hypoglycemia upon fasting, liver-specific Rab4b KO animals exhibited fasting hyperglycemia. To resolve the mechanism of hyperglycemia in Rab4b liver-specific KO animals, cell culture experiments using primary mouse hepatocytes were conducted to examine the cell autonomous effects of Rab4 KO in hepatocytes. Omitting glycerol and pyruvate from media under fasting conditions suppressed glucose production in wild-type but not Rab4b KO hepatocytes, suggesting gluconeogenesis was not required for glucose release in Rab4 KO hepatocytes. Compared to wild-type cells, Rab4 KO hepatocytes exhibited reduced glycogen levels in fasting conditions. Rab4b KO hepatocytes did not exhibit a change in glycogen phosphorylase activity or functional requirement for glucose production compared to wild-type hepatocytes in fasting conditions. However, disruption of glycophagy by inhibition of autophagosome-lysosome fusion with bafilomycin-1, or the glycophagy adaptor Stbd1, suppressed glucose production in Rab4 KO hepatocytes. Silencing of Stbd1 in animals also revealed a role for glycophagy for hypoglycemia in liver-specific Rab4b KO during fasting.

Overall, this is a very interesting and novel study. The role of the endomembrane traffic in regulating both cellular and systemic metabolism is an emerging field, with liver being at the center. This study reveals important new information that builds on prior study of Rab5 function at the early endosome in regulation of hepatic and systemic metabolism. The study uses both whole-animal models to assess physiological relevance and cell culture experiments for mechanistic study. The evidence presented for a role for glycophagy (TEM, functional studies) is compelling and provides a novel identification of a role for glycophagy in a physiological context in adult animals. Moreover, the common finding for glycophagy in both experimental models revealed by a common role for Stbd1 is a significant strength of this work. The transcriptomic studies add depth to the understanding of the physiological consequences of Rab4b deletion in animal models, and the broad range of assays used from nanostructure determination by electron microscopy to physiological measurements allows presentation of a comprehensive and internally consistent story.

There are a few minor points that can be considered:

1) Fasting causes a minor reduction of Rab4b. The mechanism uncovered here using Rab4 KO approaches reveal that Rab4 suppresses glycophagy in fasting conditions. What is the expected contribution of the (slightly) lower Rab4 levels in wild-type animals upon fasting to glucose production? Would this be the ~15% of glucose production remaining in the absence of glycerol and pyruvate seen in fasting wild-type cells in fasting conditions? This is not a suggestion to perform this experiment as the study is already very comprehensive, but to consider a brief statement to discuss this possibility.

2) The result shown in Figure 5f indicates that 4h fasting (conditions according to the figure legend) trigger a robust ~70% reduction of glycogen levels in Rab4KO hepatocytes (primary hepatocytes). Similar results shown a 60% reduction in liver glycogen in liver-specific Rab4 KO animals after 6 h of starvation. However, the hyperglycemia in Rab4 liver-specific KO animals (figure 4e) appears to continue for at least 9h. The magnitude of the reduction of glycogen levels already observed at 4-6h of fasting conditions appears inconsistent with glycophagy continuing to provide glucose for release up to and beyond 9h of fasting. Could the authors please comment if early glycophagy may be later followed by other mechanisms of

glucose release (occurring at ~6-9h in particular), or otherwise account for this apparent discrepancy?

3) What might be the mechanism by which loss of Rab4 promotes glycophagy? In the discussion, there is speculation that the role of endosomal sorting in providing membrane for phagophore expansion, and the induction of CLEAR network genes by Rab4b loss (transcriptomic data in this study) may contribute to glycophagy in Rab4 KO cells. However, these processes would appear to be permissive for general autophagy rather than specific to glycophagy. Is there a broader upregulation of general autophagy in Rab4KO cells, or does loss of Rab4 trigger a selective increase in glycophagy? It is likely that the authors have examined whether loss of Rab4b triggers a specific gain in glycophagy-specific regulators such as Stbd1, and that this does not appear to be specifically mentioned suggests that there wasn't a clear glycophagy signature exhibiting altered expression in Rab4 KO samples. If this is the case, it might be helpful to state this explicitly. Given that the amount of data presented in this manuscript is already very comprehensive and extensive, I hesitate to suggest any additional experiments, and instead suggest a brief statement to address possible mechanisms of (selective) glycophagy induction in Rab4 KO cells and animals.

Reviewer #4

(Remarks to the Author)
In this manuscript, Dussot and colleagues investigate the role of Rab4b in hepatocyte-specific metabolic regulation, particularly focusing on glycophagy—a selective autophagic process involving glycogen degradation. The authors clearly demonstrate that hepatocyte-specific depletion of Rab4b significantly alters endocytic recycling, inducing fasting hyperglycemia due to enhanced glycophagy. This enhancement is accompanied by notable metabolic reprogramming, characterized by alterations in the metabolic transcriptome and glucose metabolism, specifically manifested as fasting-induced hyperglycemia. Mechanistically, the study provides robust evidence linking Rab4b depletion to increased autophagic flux and augmented glycogen degradation via glycophagy, independent of conventional glycogenolysis pathways. The analysis of Rab4b depletion effects on endocytic recycling and metabolic transcriptomic reprogramming is adequately performed and well-supported by the data. Despite these strengths, the mechanism through which Rab4b depletion induces glycophagy remains to be further clarified for a comprehensive mechanistic understanding.To further enhance the scientific insight and comprehensive understanding of this study, the following point needs explicit addressing: Detailed elucidation of the molecular pathways and signaling cascades triggered specifically by Rab4b depletion, including direct interactions with the lysosomal/autophagic machinery, supported by experimental evidence demonstrating a mechanistic link to glycophagy.

Additional comments:
Fig. 5a, Fig. 6c, Supplementary Fig. 7b, Supplementary Fig. 8b: Molecular sizes should be shown in immunoblotting images.
Fig. 6d, Supplementary Fig. 7c: The sample numbers presented are inconsistent with those shown in the blot images.
Fig. 6g and h: Sample numbers should be clarified explicitly.

Version 1:

Reviewer comments:

Reviewer #1

(Remarks to the Author)
The authors have answered all my questions. It can be published in Nature Communications now.

Reviewer #2

(Remarks to the Author)
The authors replied to my questions sufficiently.

Reviewer #3

(Remarks to the Author)
The authors have appropriately addressed the comments resulting from the review of the initial submission.

Reviewer #4

(Remarks to the Author)
The authors have appropriately addressed not only the concerns I raised, but also those from the other reviewers. I find that the revised manuscript has been substantially improved, and I have no further comments. In my opinion, the manuscript is suitable for acceptance in its present form.

Open Access This Peer Review File is licensed under a Creative Commons Attribution 4.0 International License, which permits use, sharing, adaptation, distribution and reproduction in any medium or format, as long as you give appropriate credit to the original author(s) and the source, provide a link to the Creative Commons license, and indicate if changes were made.
In cases where reviewers are anonymous, credit should be given to 'Anonymous Referee' and the source.
The images or other third party material in this Peer Review File are included in the article's Creative Commons license, unless indicated otherwise in a credit line to the material. If material is not included in the article's Creative Commons license and your intended use is not permitted by statutory regulation or exceeds the permitted use, you will need to obtain permission directly from the copyright holder.
To view a copy of this license, visit https://creativecommons.org/licenses/by/4.0/

Point by point reply to the reviewers:

We sincerely thank the reviewers for their positive and constructive evaluation of our manuscript, highlighting the study as “very interesting”, “novel”, “well written”, and “clearly presented.” We believe that their insightful feedback, which led to the addition of new experiments substantially strengthened the manuscript and clarified the underlying mechanism by which Rab4b controls glycophagy.

Reviewer #1:

1. In Fig 1c-f, the Rab4b mRNA levels are positively correlated with blood glucose concentration. However, the manuscript showed loss of hepatic rab4b caused fasting hyperglycemia in mice. How to explain these contradicted results?

We thank the reviewer for this point, which we believe reflects a bidirectional relationship between glycemia and Rab4b expression rather than a contradiction. On the one hand, we obtained evidence that hepatic Rab4b expression is positively correlated with blood glucose levels (**Fig. 1**). Suggesting that high glucose levels induce Rab4b, whereas low glucose levels repress it. On the other hand, we discovered that Rab4b acts as a brake on glycophagy (**Fig. 6**). Taking together, these findings support a model in which, during physiological fasting, the decline in blood glucose leads to reduced Rab4b expression (Fig. 1c), thereby relieving its inhibitory effect on glycophagy. This, in turn, may contribute to maintain blood glucose levels. This mechanism is analogous to the well-established glucose-insulin loop. As glucose rise, insulin levels also increase, however insulin deficiency results in hyperglycemia. To clarify this point, we discuss this mechanism in the new version of the manuscript (**page 20**).

2. In Fig 1d-f, the author showed the mRNA levels of Rab4b are upregulated in STZ, WD or STAM mice models. The common feature of these mice models are insulin resistance or insulin deficiency-associated hyperglycemia. Are they also related to dysregulated glycophagy? The same question to the Fig 1g.

To our knowledge, no study has yet explored hepatic glycophagy in these mouse models. One study reports that myocardial accumulation of glycogen is a hallmark of hyperglycemia in mice, common to both insulin-resistant and insulin-deficient mouse models, and that this accumulation results from decreased glycophagic flux (PMID: 38484473). Nevertheless, the functional consequence of glycogen accumulation in this pathology remains unknown. In our study, we observed that Rab4b expression is elevated in hyperglycemic mice, which may suggest a reduction of glycophagic flux in the liver, as observed in the heart.

3. Rab4b controls several endocytic recycling pathways. The manuscript should reveal which protein’s endocytic recycling mediates hyperglycemia in Rab4b hepatic knockout mice after a short-time fasting.

This point is a broader concern raised by all reviewers, who requested a clearer mechanistic explanation of how Rab4b regulates autophagy/glycophagy.

The reviewer is correct that Rab4b controls several endocytic recycling pathways, including transferrin receptor recycling and the retrograde transport of the mannose-6-phosphate receptor, as we have demonstrated (PMID: 24006255; PMID: 38627612). In the previous version of the manuscript, we had not identified which pathway is necessary for Rab4b-controlled glycophagy. Here, we provide evidence that the transferrin receptor is involved in this process. Indeed, depletion of Rab4b promotes intracellular accumulation of the transferrin receptor (Fig. 2g-i), and silencing the transferrin receptor reverses the increased glucose production observed in fasted Rab4b-depleted hepatocytes.

Interestingly, a recent study demonstrates that transferrin receptor serves as a molecular platform for autophagic flux, and that intracellular accumulation of transferrin receptor is sufficient to induce autophagy (PMID: 40543506). Consistent with this, our data suggest that Rab4b depletion promotes transferrin receptor accumulation, which in turn induces autophagy/glycophagy. We incorporate these results in the new **Fig. 7g-h**, and discuss this novel mechanism in the new version of the manuscript (**page 20**).

4. In Fig 3g the author only showed the p value of these transcriptional factors. We could not tell they were upregulated or downregulated. It is better to show the fold of alternation also.

We agree that displaying fold changes is important. Accordingly, we included this new information into the revised manuscript. For some transcription factors, the activation score is below 2-fold, which cannot permit to determine whether the pathway is activated or inhibited (these are shown in grey in the graph). For the others, we implemented a color code (blue for predicted inhibition and red for predicted activation). These new results have been incorporated into the new **figure 3g**, and described in the manuscript (**page 9**).

5. The fasting blood glucose level is determined not only by insulin, but also glucagon and glucocorticoids. The author should measure these hormone levels (Fig 4c,d) in WT and Rab4b knockout mice.

We thank the reviewer for this suggestion. We measured circulating corticosterone and glucagon levels in fasted mice. Corticosterone levels were unchanged between genotypes. In contrast, glucagon levels were significantly decreased in fasted Rab4b^{HepKO} mice, likely reflecting a physiological response to the increase in glycemia. Together with the increase in insulinemia, this indicates a normal response of the pancreas. These new data have been added to the new **Supplementary Fig. 7b,c** and the new version of the manuscript (**page 10**).

6. How can Rab4b deficiency increase autophagy flux (Fig 6c)? What is the molecular mechanism? For example, does rab4b co-localize to autolysosome or autophagosome? Does rab4b affect AMPK or mTORC1 pathway?

We agree with the reviewer that identifying the underlying mechanism linking Rab4b to the increase in autophagy flux is critical, as also highlighted by the other reviewers. First as suggested by the reviewer, we assessed whether Rab4b depletion could promote autophagy by activating AMPK or inhibiting mTOR pathways under fasting conditions. Rab4b depletion did not alter AMPK phosphorylation. The phosphorylation of P70S6K, a downstream target of mTOR, was slightly increased, most likely in response to increase in insulin levels (**Fig. 7a-c**). These pathways therefore do not explain the observed increase in autophagy found in Rab4b-depleted hepatocytes. These new data have been added to the new version of the manuscript (**page 15**).

Second, we studied the co-localization of Rab4b with autolysosome or autophagosome. Due to the lack of reliable antibodies against Rab4b, we generated a knock-in mouse model expressing SnapTag-Rab4b at endogenous levels, allowing its accurate detection using an anti-SnapTag antibody. To examine the localization of SnapTag-Rab4b with autophagosomes or autolysosomes, we used the LC3-GFP-mCherry reporter. LC3-GFP-mCherry fluoresces in both green and red channels when localized to autophagosomes, whereas in autolysosomes it emits only in the red channel due to acid-dependent quenching of GFP. By performing super-resolution SSRF microscopy, we found that Rab4b shows

minimal localization to autophagosomes or autolysosomes (**Fig. 7e,f**). These new data have been added to the new version of the manuscript (**page 15**).

As detailed above (point 3), our data support a mechanism involving transferrin receptor trafficking, whose accumulation upon Rab4b depletion promotes glucose production, in agreement with a recent study showing that transferrin receptor accumulation promotes autophagy (PMID: 40543506).

7. Although the author discuss the potential clinical relevance of targeting Rab4b to glycogen storage disease (GSDs), it is better to prove it in GSD mouse model.

We agree with the reviewer that we have not demonstrated the clinical relevance of Rab4b depletion in glycogen storage disease (GSD) pathology. Following the suggestion of the reviewer #2, we tempted to generate such a model of GSD by siRNA-based depletion of the G6Pase both *in vitro* and *in vivo*, but we were unable to achieve G6Pase downregulation to obtain a blood glucose drop in control mice. Because we consider this information to be beyond the scope of the present study, and to avoid overstatement not supported by our data, we have removed this part from the Discussion.

8. There is no any Rab4b-overexpression or Rab4b rescue data throughout the manuscript. Does rab4b overexpression cause the reverse effects as compared to gene knockout?

Following the suggestion of the reviewer, we examined the effect of Rab4b overexpression on glucose production. For this we infected wildtype primary mouse hepatocytes with lentivirus containing a GFP-Rab4b construct. Despite efficient overexpression of GFP-Rab4b, in more than 80% of the cells, glucose production remains unchanged (**Supplementary figure 8**). This result underlined that Rab4b depletion and overexpression does not produce the reverse phenotype, which has already been observed for Rab proteins (PMID: 18664496 ; PMID: 29099310). We incorporated these results in the new version of the manuscript (**page 11**).

Reviewer #2:

1. In Figure 1, the authors show that hepatic Rab4b mRNA expression is decreased after 6 hours of fasting. However, they do not provide any data on Rab4b protein levels. Since protein levels may not always correlate with mRNA expression, I recommend that the authors confirm the downregulation at the protein level. Furthermore, in the discussion, the authors propose that Rab4b is involved in an early fasting-induced autophagy wave. Do they observe similar temporal dynamics at the protein level? It would be informative to examine Rab4b protein expression at multiple time points during fasting (e.g., 3, 6, 9, and 12 hours) to better characterize its regulation. In case total Rab4b levels are not changed, is its intracellular localization changed?

We agree with the reviewer that mRNA levels do not always correlate with protein levels. Due to the lack of reliable antibodies against Rab4b, we generated a knock-in mouse model expressing SnapTag-Rab4b at endogenous levels, allowing accurate Rab4b detection using an anti-SnapTag antibody. Using this approach, we found that Rab4b protein levels progressively decrease after 6 hours of fasting, reaching 50% reduction after 12 hours of fasting. We added these results in the new **Fig. 1c** and **Supplementary Fig. 2d** and in the manuscript (**page 5**).

This result suggests that the induction of glucose production by glycophagy in response to the decrease in Rab4b protein level may occur in a physiological fasting window. We discuss this possibility in the new version of the manuscript (**page 18-19**).

2. This study may have clinical implications for glycogen storage diseases, such as GSDI. Could the authors comment on whether Rab4b loss might be beneficial in the context of G6Pase deficiency? Would a dual knockdown model (e.g., siRNA-mediated suppression of both Rab4b and G6Pase) help to test this hypothesis?

We agree with the reviewer that it is an interesting perspective. However, as detailed above (see Reviewer #1 point 7) we were unable to achieve RNAi-based downregulation of G6Pase protein levels in control mice to generate a GSDI mice model. Therefore, to avoid overstatement not directly supported by our data (as underlined by reviewer #1), we decided to remove this part from the discussion.

3. All experiments appear to have been conducted in mice and primary mouse hepatocytes. To improve the translational relevance of their findings, the authors should consider replicating key results in a human-relevant model, such as human primary hepatocytes.

We agree with the reviewer that establishing the translational relevance of our findings is important. We therefore performed a RNAi-based depletion of Rab4b in human primary hepatocytes, achieving ~85% of silencing (new **Supplementary Figure 9a**). Under fasting conditions, Rab4b-depleted primary human hepatocytes showed increased glucose production (new **Supplementary Figure 9b**) and reduced glycogen content (new **Supplementary Figure 9c**), consistent with our observations in primary mouse hepatocytes. These results have been incorporated into the new version of the manuscript (**page 11**).

4. Although the authors show that Rab4b inhibits glycophagy, the underlying mechanism remains unclear. Can the authors provide further speculation or working hypotheses on how Rab4b may regulate glycophagy at the molecular level? Identifying potential receptors that might be affected by the loss of Rab4b. Is it initiation or autophagosome/lysosome fusion? This is relevant information to distinguish specific autophagic pathways, such as glycophagy in the early wave.

We agree with the reviewer that this important mechanistic information was lacking in the previous version of the manuscript, as highlighted by the other reviewers. As detailed above (Reviewer #1, points 6), our data indicate that Rab4b does not regulate autophagy through modulation of AMPK or mTOR signaling, nor through direct localization to autophagic structures. Instead, our results support a mechanism involving transferrin receptor trafficking. Indeed, Rab4b depletion leads to intracellular accumulation of the transferrin receptor (**Fig. 2**), and its silencing normalizes glucose production in Rab4b-deficient hepatocytes (**Fig. 7**). In addition, recent work shows that accumulation of the transferrin receptor promotes autophagic flux (PMID: 40543506). Together, these findings support a model in which Rab4b regulates glycophagy through control of transferrin receptor trafficking. These new data have been incorporated in the revised manuscript (**page 15)**.

Reviewer #3:

1. Fasting causes a minor reduction of Rab4b. The mechanism uncovered here using Rab4 KO approaches reveal

that Rab4 suppresses glycophagy in fasting conditions. What is the expected contribution of the (slightly) lower Rab4 levels in wild-type animals upon fasting to glucose production? Would this be the ~15% of glucose production remaining in the absence of glycerol and pyruvate seen in fasting wild-type cells in fasting conditions? This is not a suggestion to perform this experiment as the study is already very comprehensive, but to consider a brief statement to discuss this possibility.

We thank the reviewer for this insightful question. As stated above (see Reviewer#2 point 1), we now have evidence that the protein levels of Rab4b were significantly decreased after 6 hours of fasting and reached a 50 % reduction after 12 hours (new **Fig. 1c**). We agree that this physiological decrease in Rab4b protein is less pronounced than the full depletion achieved in our knockout model. However, Rab proteins are known to be highly sensitive to dosage, and partial reductions in their expression can lead to significant functional effects. Consistently, a partial loss-of-function of Rab proteins can produce phenotypes comparable to full depletion (PMID: 37924809; PMID: 26880764 PMID: 33666175). In this context, the physiological reduction in Rab4b protein levels that we observe during fasting may be sufficient to relieve its inhibitory effect on glycophagy. Whereas we cannot directly quantify the exact contribution of this decrease for glucose production during physiological fasting, our data supports a model in which Rab4b downregulation participates in the fine-tuning of glycogen mobilization during fasting. We now discuss this point in the new version of the discussion (**page 18-19**).

Concerning the question of the reviewer about the 15% of glucose production remaining in the absence of glycerol and pyruvate, we do believe that this leftover is due to the production of glucose by glycogen breakdown. Therefore, this could represent both glycogenolysis and glycophagy. We point this out in the new version of the manuscript (**page 11**).

2. The result shown in Figure 5f indicates that 4h fasting (conditions according to the figure legend) trigger a robust ~70% reduction of glycogen levels in Rab4KO hepatocytes (primary hepatocytes). Similar results shown a 60% reduction in liver glycogen in liver-specific Rab4 KO animals after 6 h of starvation. However, the hyperglycemia in Rab4 liver-specific KO animals (figure 4e) appears to continue for at least 9h. The magnitude of the reduction of glycogen levels already observed at 4-6h of fasting conditions appears inconsistent with glycophagy continuing to provide glucose for release up to and beyond 9h of fasting. Could the authors please comment if early glycophagy may be later followed by other mechanisms of glucose release (occurring at ~6-9h in particular), or otherwise account for this apparent discrepancy?

We agree with the reviewer that the apparent discrepancy between the rapid depletion of glycogen stores and the persistence of hyperglycemia over time requires clarification. First, *in vitro* conditions likely induce maximal activation of glucose production pathways, leading to an accelerated glycogen depletion compared to *in vivo* conditions. Therefore, *in vivo* data provide a more physiologically relevant framework to interpret this kinetic. We agree that after 6h of fasting the observed reduction is at two-third of the initial glycogen stores in Rab4b HepKO mice, which could be sufficient to maintain glycemia up to 9h. We acknowledge that we do not know whether the drop in glycemia observed between 9h of fasting and 24h is similar between both genotypes, or whether the drop occurs more rapidly in $Rab4^{HepKO}$ mice. We clarify this in the new version of the manuscript (**page 9**).

Given that hepatic glycogen levels were recently shown to regulate the amplitude of gluconeogenesis (PMID: 40454488), the reduced glycogen stores in $Rab4^{HepKO}$ hepatocytes may likely promote a more rapid activation of gluconeogenesis. We discuss in more details the chronological sequence of activation of gluconeogenesis, glycogenolysis, and glycophagy upon physiological situation in the new version of the manuscript (**page 18-19**).

3. What might be the mechanism by which loss of Rab4 promotes glycophagy? In the discussion, there is speculation that the role of endosomal sorting in providing membrane for phagophore expansion, and the induction of CLEAR network genes by Rab4b loss (transcriptomic data in this study) may contribute to glycophagy in Rab4 KO cells. However, these processes would appear to be permissive for general autophagy rather than specific to glycophagy. Is there a broader upregulation of general autophagy in Rab4KO cells, or does loss of Rab4 trigger a selective increase in glycophagy? It is likely that the authors have examined whether loss of Rab4b triggers a specific gain in glycophagy-specific regulators such as Stbd1, and that this does not appear to be specifically mentioned suggests that there wasn't a clear glycophagy signature exhibiting altered expression in Rab4 KO samples. If this is the case, it might be helpful to state this explicitly. Given that the amount of data presented in this manuscript is already very comprehensive and extensive, I hesitate to suggest any additional experiments, and instead suggest a brief statement to address possible mechanisms of (selective) glycophagy induction in Rab4 KO cells and animals.

We agree with the reviewer that identifying the mechanism by which Rab4b induces autophagy-dependent glycogen breakdown is pivotal, as highlighted by the other reviewers.

Our data show that autophagic flux is increased in hepatocytes lacking Rab4b (**Fig. 6c–d**), and that inhibition of autophagic flux with Bafilomycin-A1 reverses the increase in glucose production induced by Rab4b depletion (**Fig. 6e**). As discussed above (Reviewer #1, points 3 and 6; Reviewer #2, point 4), this induction of autophagy does not result from alterations in canonical autophagy signaling pathways (AMPK/mTOR). Instead, our new findings support a mechanism involving transferrin receptor trafficking. Indeed, Rab4b depletion leads to intracellular accumulation of the transferrin receptor (**Fig. 2**), and its silencing normalizes glucose production in Rab4b-deficient hepatocytes (**Fig. 7**). These findings are consistent with those reported in a recent publication showing that that transferrin receptor accumulation promotes autophagic flux (PMID: 40543506).

In addition, our results support a role for glycophagy in the observed phenotype, a process that requires prior autophagy activation, as Stbd1 depletion reversed the increase in glucose production induced by Rab4b depletion (**Fig. 6i–l**). Moreover, we observed a modest but significant increase (~20%) in GABARAPL1 expression in fasted Rab4b^{HepKO} hepatocytes (**Fig. 7a,d**), which may contribute to glycogen recruitment to the phagophore through interaction with the STBD1–glycogen complex. We also observed an increased number of autophagosomes and autolysosomes containing-glycogen, which reinforce the implication of glycophagy in glycogen breackdown.

Together, these findings support a model in which the decrease in Rab4b levels during fasting induces autophagy through regulation of transferrin receptor trafficking. This induction of autophagy during fasting, together with stabilization of Gabarapl1, promotes Stbd1-dependent glycophagy flux and enhances glucose production. These new data have been incorporated and discussed in the revised manuscript (**pages 15–16**).

Reviewer #4:

1. Detailed elucidation of the molecular pathways and signaling cascades triggered specifically by Rab4b depletion, including direct interactions with the lysosomal/autophagic machinery, supported by experimental evidence demonstrating a mechanistic link to glycophagy.

We agree with the reviewer that identifying the mechanism by which Rab4b induces autophagy-dependent glycogen breakdown is pivotal, as highlighted by the other reviewers. As detailed above (Reviewer #1, points 3 and 6 ; Reviewer #2, point 4 ; Reviewer#3, point 3), our data do not support a

role for Rab4b in the modulation of canonical autophagy signaling pathways (AMPK/mTOR) or through direct localization to autophagic structures. Instead, our results support a mechanism involving transferrin receptor trafficking. Indeed, Rab4b depletion leads to intracellular accumulation of the transferrin receptor (**Fig. 2**), and its silencing normalizes glucose production in Rab4b-deficient hepatocytes. In addition, recent work demonstrates that accumulation of the transferrin receptor promotes autophagic flux (PMID: 40543506). Together, these findings support a model in which Rab4b regulates glycophagy through control of transferrin receptor trafficking. These new data have been incorporated in the new version of the manuscript (**page 15**).

2. Fig. 5a, Fig. 6c, Supplementary Fig. 7b, Supplementary Fig. 8b: Molecular sizes should be shown in immunoblotting images.

We added the molecular size to the new version of the figures. Additionally, the original uncropped images will be provided as raw files following journal recommendations.

3. Fig. 6d, Supplementary Fig. 7c: The sample numbers presented are inconsistent with those shown in the blot images.

The reviewer is correct that the graph represents the quantification from three different blots, whereas we originally presented only one representative blot in the **figure 6d** and **Supplementary Fig 7c**. We now added all the images for all the western blots used for quantification as supplementary results (see new **Supplementary figure 10** and new **Supplementary figure 11**).

4. Fig. 6g and h: Sample numbers should be clarified explicitly.

We apologize for this missing information. We corrected accordingly.
